# Giant transposons promote strain heterogeneity in a major fungal pathogen

**DOI:** 10.1128/mbio.01092-25

**Published:** 2025-05-12

**Authors:** Emile Gluck-Thaler, Adrian Forsythe, Charles Puerner, Cecilia Gutierrez-Perez, Jason E. Stajich, Daniel Croll, Robert A. Cramer, Aaron A. Vogan

**Affiliations:** 1 Laboratory of Evolutionary Genetics, Institute of Biology, University of Neuchâtel, Neuchâtel, Switzerland; 2 Department of Plant Pathology, University of Wisconsin-Madison, Madison, Wisconsin, USA; 3 Wisconsin Institute for Discovery 145255, Madison, Wisconsin, USA; 4 Systematic Biology, Department of Organismal Biology, Uppsala University, Uppsala, Sweden; 5 Department of Microbiology and Immunology, Geisel School of Medicine, Dartmouth College 3728, Hanover, New Hampshire, USA; 6 Department of Microbiology and Plant Pathology, University of California-Riverside, Riverside, California, USA; Universidade de Sao Paulo, Ribeirao Preto, Sao Paulo, Brazil

**Keywords:** transposable element, transposon, *Aspergillosis*, *Aspergillus fumigatus*, strain heterogeneity, secondary metabolism

## Abstract

Fungal infections are difficult to prevent and treat in large part due to strain heterogeneity, which confounds diagnostic predictability. Yet, the genetic mechanisms driving strain-to-strain variation remain poorly understood. Here, we determined the extent to which *Starships*—giant transposons capable of mobilizing numerous fungal genes—generate genetic and phenotypic variability in the opportunistic human pathogen *Aspergillus fumigatus*. We analyzed 519 diverse strains, including 11 newly sequenced with long-read technology and multiple isolates of the same reference strain, to reveal 20 distinct *Starships* that are generating genomic heterogeneity over timescales relevant for experimental reproducibility. *Starship*-mobilized genes encode diverse functions, including known biofilm-related virulence factors and biosynthetic gene clusters, and many are differentially expressed during infection and antifungal exposure in a strain-specific manner. These findings support a new model of fungal evolution wherein *Starships* help generate variation in genome structure, gene content, and expression among fungal strains. Together, our results demonstrate that *Starships* are a previously hidden mechanism generating genotypic and, in turn, phenotypic heterogeneity in a major human fungal pathogen.

**IMPORTANCE:**

No “one size fits all” option exists for treating fungal infections in large part due to genetic and phenotypic variability among strains. Accounting for strain heterogeneity is thus fundamental for developing efficacious treatments and strategies for safeguarding human health. Here, we report significant progress toward achieving this goal by uncovering a previously hidden mechanism generating heterogeneity in the human fungal pathogen *Aspergillus fumigatus*: giant transposons, called *Starships*, that span dozens of kilobases and mobilize fungal genes as cargo. By conducting a systematic investigation of these unusual transposons in a single fungal species, we demonstrate their contributions to population-level variation at the genome, pangenome, and transcriptome levels. The *Starship* compendium we develop will not only help predict variation introduced by these elements in laboratory experiments but will serve as a foundational resource for determining how *Starships* impact clinically relevant phenotypes, such as antifungal resistance and pathogenicity.

## INTRODUCTION

Infectious diseases caused by fungi pose a grave threat to human health and society. The World Health Organization recently coordinated a global effort to prioritize research among fungal pathogens based on unmet research needs and public health importance (1). Among the pathogens deemed most important for research include *Aspergillus fumigatus*, a globally distributed opportunistic human pathogen causing disease in an estimated 4 million people yearly (2, 3). Several infectious diseases are caused by *A. fumigatus*, including invasive pulmonary aspergillosis, which manifests primarily in immunocompromised individuals with mortality rates of up to 85% (2, 4). The treatment of *A. fumigatus* is complicated by its remarkable variation in virulence, resistance to antifungals, metabolism, and other infection-relevant traits (3, 5–8). Strain heterogeneity confounds “one size fits all” therapies and poses a significant challenge for developing efficacious disease management strategies (9). Recent evaluations of the *A. fumigatus* pangenome have revealed extensive genetic variability underlying variation in clinically relevant traits, such as antifungal resistance and virulence, yet in many cases, the origins of such variation remain unexplained (10–12). Determining the genetic drivers of strain heterogeneity will help accelerate the development of strain-specific diagnostics and targeted therapies.

Mobile genetic elements (MGEs) are ubiquitous among microbial genomes, and their activities profoundly shape the distribution of phenotypic variation (13). MGE transposition generates structural variation that directly impacts gene regulation and function (14–16), and many elements also modulate genome content by acquiring genes as “cargo” and transposing them within and between genomes, facilitating gene gain and loss (17, 18). The ability to generate contiguous genome assemblies with long-read sequencing technologies has dramatically enhanced our ability to find new lineages of MGEs (13, 19). For example, we recently discovered *Starships*, a new superfamily of MGEs found across hundreds of filamentous fungal taxa (20–22). *Starships* are fundamentally different from other fungal MGEs because they are typically 1–2 orders of magnitude larger (~20–700 kb versus 1–8 kb) and carry dozens of protein-coding genes encoding fungal phenotypes (23). In addition to flanking short direct repeats, all *Starships* possess a “captain” tyrosine site-specific recombinase at their 5′ end that is both necessary and sufficient for transposition (21). Several *Starships* mediate the evolution of adaptive phenotypes: for example, *Horizon* and *Sanctuary* carry the ToxA virulence factor that facilitates the infection of wheat, and *Hephaestus* and *Mithridate* encode resistance against heavy metals and formaldehyde, respectively (24–27). *Starships* have been experimentally shown to horizontally transfer between fungal species (28), and an increasing number of comparative studies implicate them in the horizontal dissemination and repeated evolution of diverse traits (20, 21, 25–27). *Starships* may also fail to re-integrate into the genome during a transposition event, leading to the loss of the element, its cargo, and its encoded phenotypes (21). Through horizontal transfer and failed re-integration events, *Starships* contribute directly to the generation of rapid gene gain and loss polymorphisms across individuals. However, we know little about how *Starships* drive genetic and phenotypic variation in fungal species relevant for human health and disease.

Here, we conduct the first systematic assessment of *Starship* activity and expression in a human fungal pathogen to test the hypothesis that these unusual transposons are a source of strain heterogeneity. We reveal that *Starships* are responsible in part for generating key instances of previously unexplainable variation in genome content and structure. Our interrogation of 508 diverse clinical and environmental strains of *A. fumigatus*, combined with highly contiguous assemblies of 11 newly sequenced strains, enabled the unprecedented quantification of *Starship* diversity within a single species and even within the same “reference” strain. We leveraged the wealth of functional data available for *A. fumigatus* to draw links between *Starship*-mediated genetic variation and phenotypic heterogeneity in secondary metabolite and biofilm production contributing to pathogen survival and virulence. We analyzed multiple transcriptomic studies and determined that variation in *Starship* cargo expression arises from strain- and treatment-specific effects. By revealing *Starships* as a previously unexamined mechanism generating phenotypic variation, our work sheds light on the origins of strain heterogeneity and establishes a predictive framework to decipher the intertwining impacts of transposons on fungal pathogenesis and human health.

## RESULTS AND DISCUSSION

### Data curation and long-read sequencing

*Starships* are difficult (but not impossible) to detect in short-read assemblies due to their large size and frequent localization in regions with high repetitive content (20). The wealth of publicly available short-read genomes is still of high value, however, due to the breadth of sampling they provide, combined with the still relevant possibility of finding *Starships*, especially in higher quality short-read assemblies, such as those available for *A. fumigatus*. We, therefore, deployed a hybrid sampling strategy that leveraged both short- and long-read genome assemblies, combined with short-read mapping to genotype *Starship* presence/absence, to enable an accurate and precise accounting of how *Starships* impact strain heterogeneity. We downloaded 508 publicly available *A. fumigatus* assemblies (mostly assembled from short-read data) that span the known genetic diversity of this species isolated from environmental and clinical sources (51.7% clinical, 47.9% environmental, 0.4% unknown isolation source) and used Oxford Nanopore to sequence 11 additional strains with long-read technology (66.7% clinical, 25% environmental, 8.3% unknown) for a combined total of 519 assemblies (10, 11). The 11 isolates selected for long-read sequencing were chosen because they represent commonly utilized laboratory strains, clinical isolates, and environmental strains from three major *A. fumigatus* clades (10). We performed additional short-read Illumina sequencing of the 11 strains to provide additional support and error-correct the long-read assemblies. The long-read assemblies are of reference quality, representing nearly full chromosome assemblies with an L50 range of 4–5 and an N50 range of 1.9–4.7 Mb, and are thus ideal complements to the available short-read assemblies for investigating the connection between *Starship*s and strain heterogeneity (Table S1).

### At least 20 active and distinct *Starships* vary among *A. fumigatus* strains

We began evaluating the impact of *Starships* on strain heterogeneity by systematically annotating them in the 519 assemblies using the starfish workflow (22). We identified a total of 787 individual *Starships* and validated these predictions by manually annotating a subset of 86 elements (Tables S2 and S3; Materials and Methods) (10, 11). As expected, more *Starships* were recovered in total from short-read assemblies, but on average, more *Starships* were recovered from each reference-quality long-read assembly, highlighting the utility of sampling both short- and long-read assemblies (Fig. S1). Importantly, unlike many other fungal transposons, we found that with few exceptions, if a *Starship* is present in a strain, it is present at most in a single copy. Some other species appear to readily accumulate multiple copies of the same *Starship* (20), but this is not the case for *A. fumigatus*. In other fungi, genome defense mechanisms, such as repeat-induced point mutations, are capable of degrading multi-copy *Starships* and may act to limit their copy number expansion (21); however, it remains unclear whether such mechanisms also explain the lack of copy number variation observed in *A. fumigatus*.

**Fig 1:**
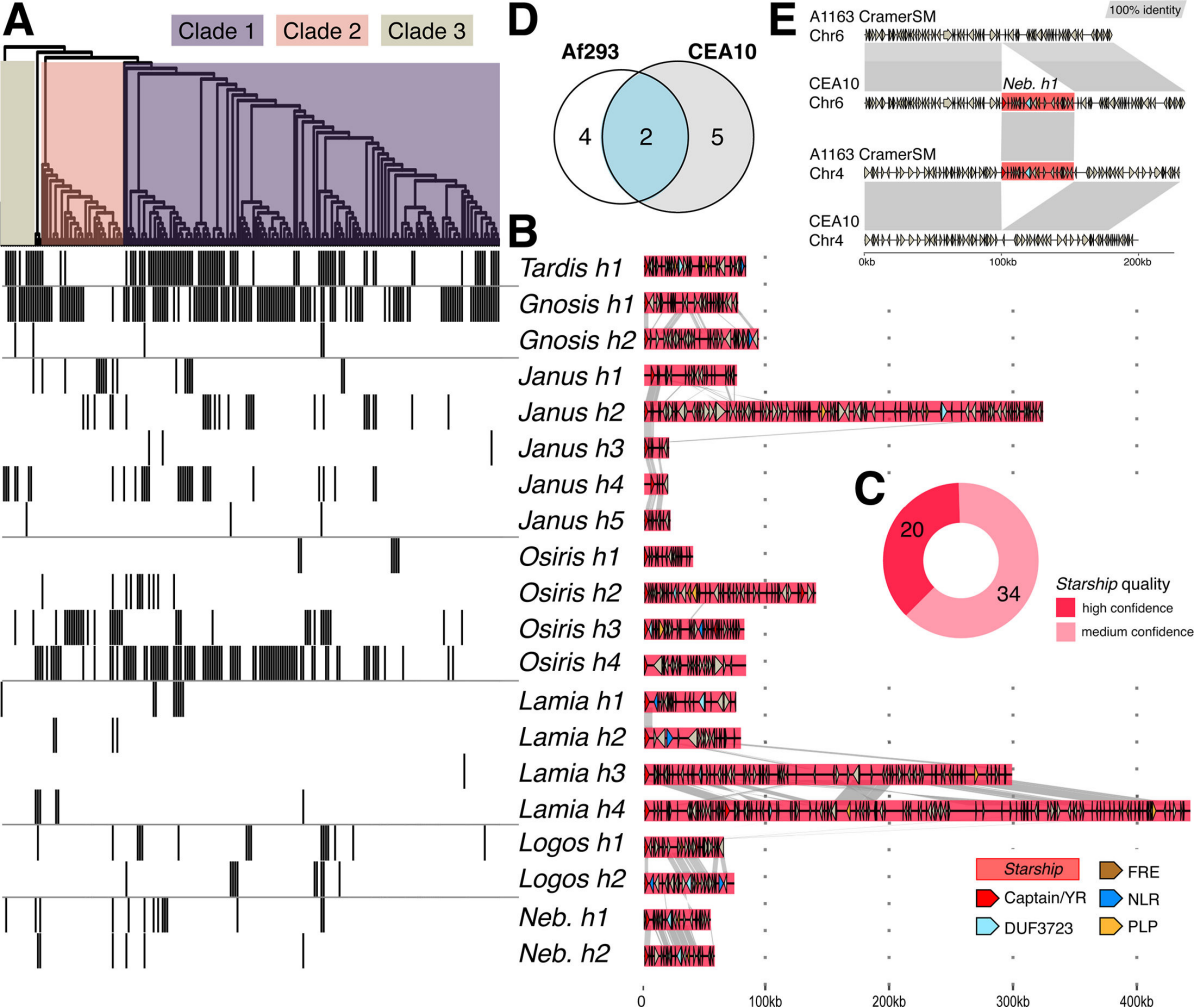
At least 20 distinct *Starships* carrying hundreds of protein-coding genes vary in their presence/absence across *Aspergillus fumigatus* strains. (A) Top: an SNP-based maximum likelihood tree of 220 *A*. *fumigatus* strains from reference 10 (visualized as a cladogram for clarity) and color-coded according to phylogenetic clade as defined by reference 10. Bottom: a heatmap depicting the presence (gray) and absence (white) of 20 high-confidence *Starships* in each isolate. (B) Schematics and sequence alignments of the type elements from 20 high-confidence *Starships*, where links between schematics represent alignable regions ≥500 bp and ≥80% nucleotide sequence identity, and arrows represent predicted coding sequences. (C) A donut chart summarizing the number of distinct types of *Starships* by the quality of their prediction. (D) A Venn diagram indicating the number of shared and unique high-confidence *Starships* in the reference strains Af293 and CEA10 (Table S7). (E) Pairwise genome alignments between the CEA10 isolate from BioSample SAMN28487501 and the CEA10-derived A1163 isolate sequenced in this study demonstrating *Starship* movement. *Nebuchadnezzar h1* is present on chromosome 6 in CEA10 (CP097568.1: 3645813–3698867) and absent at the corresponding locus in A1163 (NODE10: 188873–188877). Conversely, *Nebuchadnezzar h1* is present on chromosome 4 in A1163 (NODE6: 77737–130777) but absent from the corresponding locus in CEA10 (CP097566.1:1344957–1344961).

To determine how many different *Starships* actively generate variability in *A. fumigatus*, we grouped *Starships* into discrete types following a hierarchical framework that incorporates amino acid sequence comparisons of captain tyrosine recombinases and nucleotide sequence comparisons of the entire element (23). Following Gluck-Thaler and Vogan, we assigned each *Starship* element to a family (based on similarity to a reference library of captain tyrosine recombinases), a navis (Latin for “ship”; based on orthology among the *A. fumigatus* captains), and a haplotype (based on k-mer similarity scores of the cargo sequences; Materials and Methods). For example, *Starship Osiris h4* belongs to haplotype 4 within the *Osiris* navis, which is part of the Enterprise family. The most reasonable conclusion when observing the same transposon bounded by identical direct repeats at two different loci in two individuals is that this transposon is presently or recently active (21). Using a threshold that required observing the same navis-haplotype (hereafter *Starship*) at two or more sites, we identified 20 high-confidence *Starships* that met these criteria and 34 medium-confidence *Starships* that did not. These data reveal a phylogenetically and compositionally diverse set of *Starships* actively transposing within *A. fumigatus* that together make up this species’ *Starship* compartment (Materials and Methods, Table 1, Fig. 1, Tables S4 and S5) (23). For each of the 20 high-confidence elements, we aligned representative copies present in different genomic regions to highlight how precisely and repeatedly element boundaries are conserved across transposition events (Fig. S2).

**TABLE 1:** High-confidence *Starships* in *Aspergillus fumigatus*^*a*^

Family	Name	Predicted target site	Mean length in bp (SD)	Type element	Functions of interest in type element	Reference strains
Tardis	*Tardis h1*	TACGGAGTAG	81,743 (9,347)	W72310-lr_s00161	GH71, SAH	Af293
Prometheus	*Gnosis h1*	A(N_3_)CTA(N_17_)T	82,313 (23,570)	A-fum-AFUG-100413-0667_s00261	GH18, lysM	Af293, CEA10
Prometheus	*Gnosis h2*	n.d.	93,292 (60)	CM2733_s04518	bafY, fumihopaside A BGC	CEA10
Phoenix	*Janus h1*	n.d.	62,365 (19,076)	F14513G_s04800	CHROMO	n.d.
Phoenix	*Janus h2*	A(N_3_)TTACTA (N_2_)A(N_15_)CT	217,821 (102,982)	47-4_s00005	CHROMO, DHDPS, GH18, GH71	CEA10
Phoenix	*Janus h3*	A(N_3_)TTACTA (N_2_)A(N_15_)CT	42,070 (37,412)	SF1S6_s05434	CHROMO	n.d.
Phoenix	*Janus h4*	n.d.	19,097 (5,047)	CM6458_s04616	CHROMO	n.d.
Phoenix	*Janus h5*	n.d.	34,546 (18,524)	47-10_s00011	CHROMO	n.d.
Enterprise	*Osiris h1*	5S rDNA	37,633 (2,148)	A-fum-AFIS-13708-CDC-14_s00209	Putative BGC	n.d.
Enterprise	*Osiris h2*	5S rDNA	138,260 (2,424)	B5269_s04148	ARS, B-LAC, COP	n.d.
Enterprise	*Osiris h3*	5S rDNA	70,476 (28,148)	Afu-218-E11_s00395	bafB, hrmB	CEA10
Enterprise	*Osiris h4*	5S rDNA	51,342 (13,227)	Af293_s00032	Putative BGC	Af293
Galactica	*Lamia h1*	A(N)TAGT	69,899 (24,972)	S02-30_s00148	GH18, lysM	n.d.
Galactica	*Lamia h2*	A(N)TAGT	65,414 (6,136)	ATCC42202_s00060	Fumigermin BGC	n.d.
Galactica	*Lamia h3*	A(N)TAGT	236,680 (74,695)	ISSF-21_s05253	GH18, GH71	n.d.
Galactica	*Lamia h4*	A(N)TAGT	291,189 (215,918)	F7763_s04695	bafX, fumigermin BGC, GH3, GH18, GH71, GT8, GT31	Af293
Prometheus	*Logos h1*	A(N_10_)TACTTATTA(N)A(N_6_)A	69,008 (5,313)	CEA10_s00024	bafZ	CEA10
Prometheus	*Logos h2*	n.d.	56,189 (31,755)	CEA10_s00031	bafC	CEA10
Hephaestus	*Neb h1*	TTACA(N_5_)AAT	48,346 (5,585)	AF293_s00037	ARS, bafA, cgnA, hrmA	Af293, CEA10
Hephaestus	*Neb h2*	TTACA(N_5_)AAT	54,751 (7,226)	AF293_s00031	ARS	Af293

^*a*^ Abbreviations: GH: glycosyl hydrolase; GT: glycosyl transferase; SAH: salicylate hydroxylase; DHDPS: dihydrodipicolinate synthetase; CHROMO: chromatin modification domain; ARS: arsenic resistance cluster; COP: copper resistance; B-LAC: beta-lactamase; n.d.: could not be determined.

After using all available genome assembly data to define the *Starship* compartment with automated methods, we explored a variety of other approaches for augmenting and validating our predictions, including manual annotation, BLAST-based detection, and short-read mapping (Materials and Methods). Certain detection methods were more appropriate for particular analyses, resulting in the partitioning of our findings into the high-confidence, the expanded, and the genotyping data sets. To analyze *Starship* features like gene content and pangenome distributions (see below), we limited our analyses to the set of 459 high-confidence elements for which we have end-to-end boundaries and evidence of transposition (high-confidence data set). For analyses that examine the presence/absence of elements across genomic regions, we used an expanded set of 1,818 elements that consists of the 787 high- and medium-confidence elements, as well as 1,031 elements whose presence was detected through BLAST searches, because these methods maximize our ability to correctly predict the presence/absence of an element in a given region (expanded data set; Tables S4 and S5). For genotyping analyses that focus on detecting the presence/absence of an element, regardless of where it is found in the genome, we took a conservative approach by limiting our analysis to 13 reference quality assemblies (reference strains CEA10 and Af293 in addition to the 11 newly sequenced long-read strains) plus 466 *A*. *fumigatus* strains with publicly available paired-end short-read Illumina data that were used to validate *Starship* presence/absence by short-read mapping to a reference *Starship* library (genotyping data set; Table S21).

### *Starship* distributions are partially explained by strain relatedness but overall heterogeneous

All high-confidence *Starships* show polymorphic presence/absence variation, representing a previously unexamined source of genetic heterogeneity across *A. fumigatus* strains (Fig. 1A, Table S6). For example, the two commonly used reference strains Af293 and CEA10 carry 11 different high-confidence *Starships* but share only four in common (or only two, using a more conservative threshold of not counting fragments or degraded elements; Fig. 1D, Table S7). The number of high-confidence *Starships* per long-read isolate ranges from 0 to 11 (median = 3, standard deviation [SD] = 3.39) (29, 30). To identify the underlying drivers of variation in *Starship* distributions, we investigated the relationship between *Starship* repertoires and strain relatedness by testing if phylogenetic signal underlies *Starship* distributions. We genotyped *Starship* presence/absence using short-read mapping in the best sampled clade of *A. fumigatus* with well-resolved phylogenetic relationships (Clade 1 *sensu* [10], *n* = 151) and found that isolate relatedness is weakly but significantly correlated with similarity in *Starship* repertoire, which is indicative of weak phylogenetic signal in *Starship* distributions (Fig. S3; Table S8, Mantel r = 0.163; *P* = 0.001, using the genotyping data set). The relatively weak but significant correlation indicates that although closely related isolates tend to share similar *Starships*, relatedness alone cannot explain all observed variation in *Starship* repertoires. Thus, we conclude *Starships* are a source of genetic heterogeneity among even closely related strains.

While no sequence-based or phylogenetic method to our knowledge would enable us to unequivocally differentiate horizontal transfer events from incomplete lineage sorting, loss, or sexual and parasexual recombination at the within species level, we hypothesize that horizontal transfer within *A. fumigatus* is at least partially responsible for generating the observed patchy distributions of *Starships. Starship* horizontal transfer has occurred repeatedly between individuals of different genera, and barriers to transfer are likely lower within species (25, 26). *Starship* horizontal transfer has recently been experimentally demonstrated under simple co-culture conditions between *A. fumigatus* and *Paecilomyces variotii*, which are separated by approximately ~100 million years of evolutionary divergence (28). Surveys of the *Paecilomyces* fungi further estimate that ~1/3 of *Starships* in this genus has evidence of horizontal transfer in natural populations (28). The dynamics of *Starship* horizontal transfer among *A. fumigatus* strains must be determined with future laboratory experiments.

### *Starships* actively transpose in isolates of the same laboratory strain

We examined the potential for *Starships* to introduce unwanted variation into laboratory experiments by comparing isolates of the same reference strain used by different research groups. First, we found that *Starship* insertions in the reference Af293 assembly used in our study coincide with 4/8 putative structural variants inferred in other Af293 strains using RNAseq expression data by Collabardini et al. (Table S9). This suggests that in addition to other mechanisms, *Starship* transposition and/or loss contributes to structural variation observed among isolates of what should otherwise be clones of the same strain (6).

We gathered additional evidence supporting our hypothesis by comparing independently collected DNA sequencing data from different isolates of the same or derived strains. We compared the locations of *Starships* between the long-read CEA10 = CBS144.89 reference assembly (31) and six other CEA10 and CEA10-derived isolates from various labs, including our own, using short-read mapping and de-novo assembly comparisons (Materials and Methods). We found that *Starship Nebuchadnezzar h1* has jumped from chromosome 6 in the long-read reference CEA10 and A1160 assemblies and some A1163 strains to chromosome 4 in the A1163 isolate used by our lab (A1163-Cramer_SM) likely at some point after we acquired this isolate from its original culture collection stock (Fig. 1E). A1163 is a commonly used laboratory strain derived from CEA10; the only differences between A1163 compared with CEA10 should be that its native *pyrG* has a nonsense mutation and that it harbors an ectopic insertion of the *Aspergillus niger pyrG* (32). Similarly, we evaluated *Starship* heterogeneity in three additional strains, namely, S02-30 (=AF100-9B), TP9 (=08–19-02-30) and ATCC46645, that met the criteria of having independently collected DNA sequencing data sets and long read assemblies. We found that ATCC46645 showed signs of *Starship* heterogeneity among isolates sequenced by different research groups. Specifically, a small number of reads supported precise excision events for *Nebuchadnezzar h1*, suggesting this *Starship* has transposed or has been lost in a subset of the nuclei within the short-read sequenced isolate (Fig. S4). This suggests that some *Starships* appear to be active under laboratory conditions, which is directly relevant for experimental design in this case, as *Nebuchadnezzar h1* carries the HAC gene cluster known to impact biofilm-associated virulence (see below), and genes on *Starships* may be lost through failed transposition events (21, 33).

Together, *Starship*-mediated variation among isolates of the same “clonal” *A. fumigatus* strain suggests these transposons cause genomic instability over short-enough timescales to potentially impact routine laboratory work and experimental reproducibility. It is interesting to note that not all strains or closely related strains demonstrate intraspecific variation: for example, although we did not detect variation in *Starship* location or presence/absence between the long-read CEA10 and A1160 assemblies, which have been separated for decades (31), we did detect variation between this CEA10 strain and the A1163 isolate that we routinely use in our lab, indicating that much remains to be known about the signals and triggers promoting within-strain *Starship* variation.

### *Starships* mobilize upwards of 16% of the accessory genome that differs across strains

**Fig 2:**
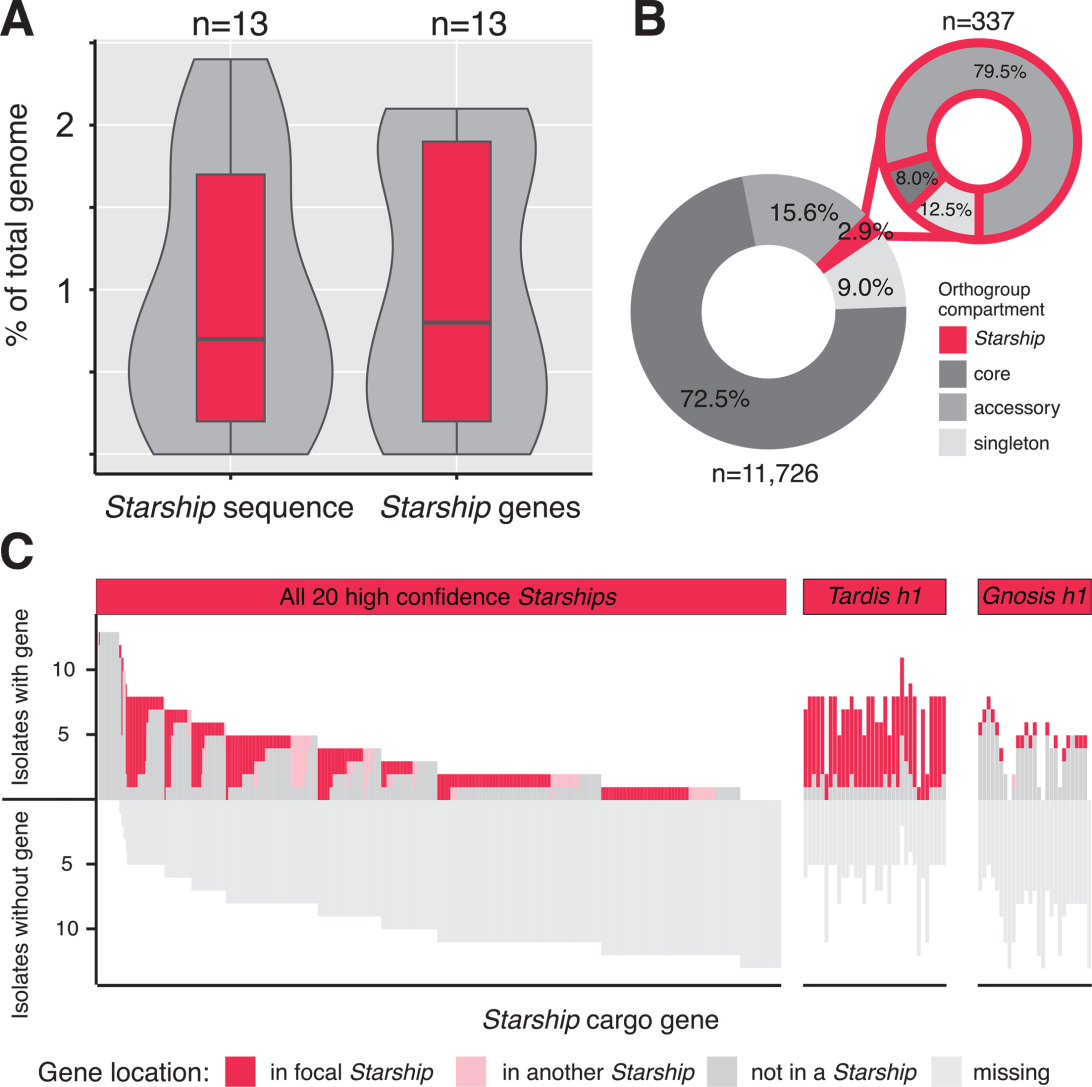
*Starships* are enriched in accessory genes whose presence/absence and genomic location vary across *Aspergillus fumigatus* strains. All panels visualize data derived from 13 reference-quality *A. fumigatus* assemblies and the 20 high-confidence *Starships* (*n* = 459 elements total). (A) Box-and-whisker plots summarizing the total percentage of nucleotide sequence and predicted genes carried by *Starships* per genome. (B) Donut charts summarizing the percentages of gene orthogroups in the core, accessory, singleton, and *Starship-*associated compartments of the *A. fumigatus* pangenome (Table S10). (C) Iceberg plots summarizing the genomic locations of the single best BLASTp hits (≥90% identity, ≥33% query coverage) to the cargo genes from the type elements of all 20 high-confidence *Starships* (left) and two individual *Starships* (right; Table S12). Each column in the iceberg plot represents a cargo gene and is color-coded according to the genomic location of hits (full data set in Fig. S5).

*A. fumigatus* strains differ extensively in the combinations of genes found in their genomes, resulting in a large and diverse pangenome (10, 11). To quantify how much pangenomic variation is attributable to actively transposing *Starships*, we estimated the total proportion of genomic content present in the 20 high-confidence *Starships* (Fig. 2; Materials and Methods). We examined these *Starships* in the 13 reference-quality assemblies and found that between 0 and 2.4% of genomic nucleotide sequence (and 0–2.1% of all genes per genome) is mobilized as *Starship* cargo (Fig. 2A). We then built a pangenome with all 519 strains and extracted all orthogroups (i.e., genes) found in the 13 reference-quality genomes to gain insight into the distribution of *Starship*-associated gene content (Table S10). Across the 13 isolate pangenome, 2.9% of all genes, which correspond to 9.7% of all accessory and singleton orthogroups, have at least one member carried as *Starship* cargo (Fig. 2B). Accessory and singleton orthogroups are overrepresented >threefold in *Starships*, with ~92% of *Starship*-associated orthogroups being either accessory or singleton compared with 24.6% of non-*Starship* associated orthogroups. We determined the upper bounds of this conservative estimate by examining the 13 isolate pangenome in the context of all 54 high- and medium-confidence *Starships* and found that 4.8% of all orthogroups, representing 16% of all accessory and singleton orthogroups, have at least one member carried as *Starship* cargo (Fig. S5; Table S11). Drawing parallels from decades of observations of bacterial MGEs (17) and recently published experimental data from *Starships* (21, 28), we hypothesize that localization on a *Starship* promotes a gene’s rate of gain and loss through *Starship-*mediated horizontal transfer and failed re-integration events (21). Together, these data reveal a previously hidden association between *Starships* and the making of *A. fumigatus*’ accessory pangenome.

### *Starships* differ in their potential to mediate gain and loss of unique sequences.

We further determined the potential for *Starships* to introduce variation into *A. fumigatus* strains by testing if *Starship* genes are uniquely found in those elements or found elsewhere in the genome. We calculated the degree of association between a given cargo gene and a *Starship* by identifying the genomic locations of best reciprocal BLAST hits to genes in the 20 high confidence-type elements (Fig. 2C; Fig. S6; Tables S12 and S13; Materials and Methods). Across the 13 reference-quality assemblies, we found that the vast majority of *Starship* genes display presence/absence variation between strains (i.e., are accessory or singleton), although several genes are present in conserved regions in these particular strains (while seemingly counter-intuitive, these genes do vary in their presence/absence and are *Starship-*associated when examining the larger 519-strain population, as expected). We found that many genes are almost always carried as cargo in active *Starships* when present in a given genome (e.g., the majority of cargo on *Tardis h1*), while others have weaker associations and are found in both *Starship* and non-*Starship* regions across different strains (e.g., *Gnosis h1*). Variation in the degree to which genes associate with active *Starships* suggests elements differ in capacity to generate accessory sequence variation among strains, highlighting the importance of investigating *Starships* at the individual element level.

### *Starship* activity generates structural variation at a genome-wide scale

**Fig 3:**
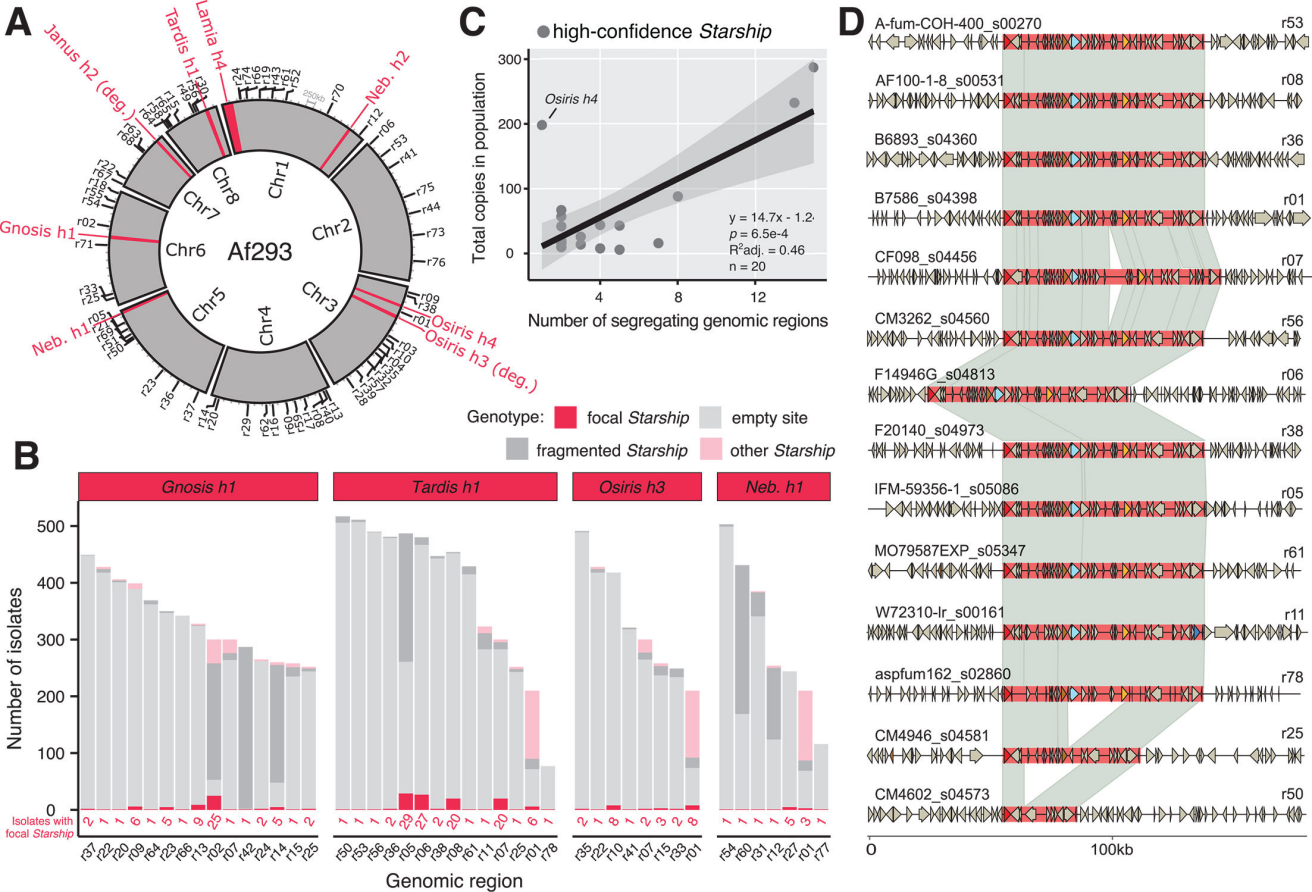
*Starships* and their insertion sites are distributed across all major chromosomes in the *Aspergillus fumigatus* genome. (A) A Circos plot summarizing all inserted *Starships* (in red) and all genomic regions containing either an empty insertion site or a fragmented *Starship* (in black, along perimeter and labeled with the *r* prefix) in the eight chromosomes of the *A. fumigatus* reference strain Af293 (Table S14). All genomic regions contain a *Starship* insertion in some other individual from the 519-strain population. (B) Barcharts summarizing the genotypes of segregating genomic regions associated with the four most active high-confidence elements in the 519-strain population (Table S16; full data set in Fig. S7). If an isolate did not have any *Starships* within a given region, it was assigned either an “empty” or “fragmented” genotype (Materials and Methods). (C) A scatterplot summarizing the relationship between the number of genomic regions containing a given *Starship* and the total number of copies of that *Starship* in the 519-strain population, where each point represents one of the 20 high-confidence *Starships.* A line derived from a linear regression model is superimposed, with shaded 95% confidence intervals drawn in gray. (D) Alignments of *Tardis h1* copies ± 50 kb of flanking sequence across 14 genomic regions. Links between schematics represent alignable regions ≥1,000 bp and ≥90% nucleotide sequence identity.

We next asked where *Starship*-mediated variation occurs in the genome to better understand the implications of *Starship* activity for genome organization. We identified the genomic locations where *Starships* introduce structural variation by sorting all 787 high- and medium-confidence elements, along with elements detected by BLASTn, into homologous genomic regions (Tables S14 to S16; expanded data set; Materials and Methods). This enabled us to genotype individuals in the 519-strain population for segregating *Starship* insertions that are polymorphic across strains (Fig. 3; Fig. S7; Materials and Methods). Across all strains and chromosomes, we found 79 regions that contain at least one segregating “empty” insertion site, for a total of 154 sites distributed across all eight chromosomes (a single region can have >1 insertion site if they are located close to each other). The average number of empty sites per genome is 44.5 (range = 9–63, SD = 13.88), indicating that each strain harbors dozens of sites with structural variation introduced by *Starships*. For example, the Af293 reference strain has a total of six full-length *Starships* with annotated boundaries and two *Starship* fragments and a total of 56 segregating empty sites (Fig. 3A). We found a significant linear relationship between the number of genomic regions a given *Starship* is present in (a proxy for transposition activity) and the total copy number of that element in the 519-strain population, suggesting active *Starships* contribute more to strain heterogeneity compared with less active elements (Fig. 3C; *y* = 14.7*x* − 1.24; *P =* 6.5e^−4^; *R*^2^ adj = 0.46; expanded data set). Predicted *Starship* boundaries are precisely conserved across copies present in different genomic regions, suggesting transposition, and not translocation or segmental duplication, is the major mechanism responsible for generating variation in *Starship* location (Fig. 3D).

Given that *Starships* are mobilized by a tyrosine site-specific recombinase (21), we attempted to predict each *Starship*’s target site to gain insight into the genomic sequences that are susceptible to *Starship* insertions. We manually curated the sequence features of a large sample of high-confidence *Starships* and found 19/20 have identifiable direct repeats (DRs) ranging from 2 to 12 bp in length, while 18/20 have terminal inverted repeats (TIRs; Table 1, Table S3). DRs typically reflect a portion of the element’s target site, while TIRs are predicted to facilitate transposition (34). While precise target site motifs must be confirmed experimentally, the target site TTACA(N_5_)AAT that we recovered for *Nebuchadnezzar* elements, which belong to the Hephaestus family, resembles the canonical *Hephaestus* target site TTAC(N_7_)A, demonstrating the utility of our approach as a first step toward understanding what sequence features promote the gain and loss of *Starship*-mediated variation (21).

Generally, DRs > 6 bp are associated with targeting of the 5S rDNA gene (e.g., *Osiris*) presumably because this is a relatively stable target site (22). In contrast to this, *Tardis* has a highly conserved 9–11 bp DR that does not correspond to the 5S gene. A k-mer analysis shows that the 10 bp consensus motif CTACGGAGTA is strongly overrepresented in the genome of Af293 (>99.99th percentile of k-mers; Fig. S8), indicating that this motif may represent some other type of highly conserved genomic sequence, such as a transcription factor binding site. This motif overlaps with predicted motifs associated with C6 zinc cluster factors in *A. nidulans* (https://jaspar.elixir.no/matrix/UN0291.2/) (35), and this DR sequence is further conserved among other *Eurotiomycetes* (22); however, the exact functions of these predicted motifs must be confirmed experimentally. This result highlights the fact that studying *Starship* elements in detail can reveal other important aspects of a species’ biology, and characterizing the *Tardis* motif is of interest for future research.

### *Starships* mediate variation at an idiomorphic biosynthetic gene cluster

**Fig 4:**
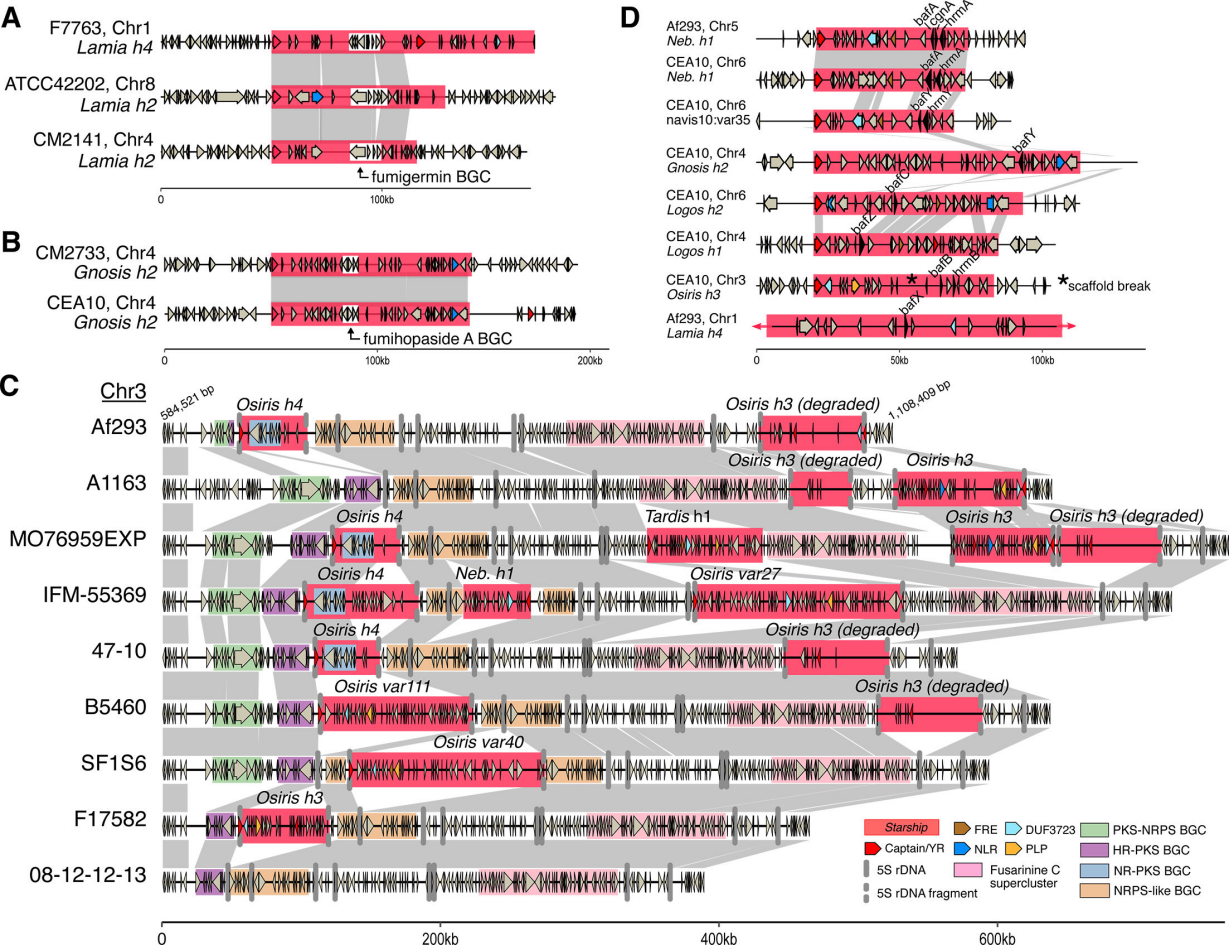
*Starships* mobilize adaptive traits and generate allelic diversity among *Aspergillus fumigatus* strains. Schematics and alignments. (A) *Starship Lamia h2* and *h4*, which carry the biosynthetic gene cluster (BGC) encoding the polyketide secondary metabolite fumigermin (36), shown inserted at three independent sites. (B) *Starship Gnosis h2* carrying the BGC encoding the terpene secondary metabolite fumihopaside A (37), shown inserted at two independent sites. (C) A large region on chromosome 3 previously identified as an idiomorphic BGC (38) containing multiple segregating *Starship* insertions and various combinations of putative BGCs, including a non-reducing polyketide synthase (NR-PKS) BGC carried by *Starship Osiris h4. Starships* from the *Osiris* navis specifically insert in the 5S rDNA sequence and are predicted to fragment it. (D) Eight *Starships* in the reference strains Af293 and CEA10 that all carry homologs of *biofilm architecture factor A* (*bafA*) (33). *Starship Nebuchadnezzar h1* (*Neb. H1*) carries *bafA* as part of the H_A_AC (*hrmA-*associated gene cluster), while *Starship* navis*10-var35* carries *bafB* as part of H_B_AC, and *Starship Osiris h3* carries *bafC* as part of H_C_AC (33). Only a portion of *Starship Lamia h4* is visualized for figure legibility. All data for panels A–D were collected from the 519 *A. fumigatus* strain population (Table S5). Links between schematics represent alignable regions ≥5,000 bp and ≥95% nucleotide sequence identity, and arrows represent predicted coding sequences. Abbreviations: polyketide synthase (PKS); non-ribosomal peptide synthetase (NRPS); highly reducing (HR).

Several genomic regions harbor multiple types of *Starships*, raising the possibility that strain heterogeneity arises in part from the formation of *Starship* insertion hotspots. We investigated the genomic region with the highest density of *Starship* insertions to define the upper bounds of *Starship*-mediated structural variation at a single locus (Fig. 4C; Materials and Methods). This region spans an average of 498.76 kb (range = 158.07–781.54 kb; SD = 83.02 kb) across the *n* = 210 strains for which we could detect it and ranges from position 584,521 to 1,108,409 on chromosome 3 in the Af293 reference assembly (representing 12.84% of the entire chromosome). By supplementing starfish’s automated genotyping with manual annotation of nine strains with distinct alleles at this region, we found this region contains at least seven distinct *Starships* with identifiable DRs and one degraded *Starship* that range from 47.52 to 151.62 kb long and are inserted into six independent segregating sites (Fig. 4C). The majority (75%) of the *Starships* in this region are inserted into 5S rDNA coding sequences, which effectively fragments that copy of the 5S rDNA gene. Total 5S rDNA copy number varies between 28 and 33 in the 13 reference quality assemblies (median = 31, SD = 1.4; Table S17), but between six and eight intact copies are typically present at this single locus, representing a ~10-fold enrichment of the 5S rDNA sequence relative to background expectations given the length of this region (using 5S rDNA frequencies in the Af293 genome). Thus, the enrichment of 5S rDNA at this locus potentiates *Starship*-mediated variation and the generation of a *Starship* hotspot.

We were surprised to find that in addition to multiple segregating *Starships*, the chromosome 3 region contains an idiomorphic biosynthetic gene cluster (BGC) that was previously noted to be a recombination hotspot (Cluster 10 *sensu* [38, 39]). The idiomorphic BGC locus is polymorphic for upwards of four smaller BGC modules and is upstream of a fifth BGC encoding the biosynthesis of fusarinine C (BGC8 *sensu* [40]) that itself is embedded within a larger stretch of sequence containing NRPS and KR-PKS core genes. While the idiomorphic modules were not predicted by antiSMASH, manual inspection revealed that each contains a different core SM biosynthesis gene and numerous other genes involved in metabolic processes, suggesting they are part of a larger BGC or represent different cryptic BGCs. The NR-PKS BGC module at this locus is carried by *Starship Osiris h4*, and its presence/absence is directly associated with the presence/absence of the element, implicating *Starships* as a mechanism generating idiomorphic BGCs. Furthermore, some BGC modules are disrupted by the insertion of a *Starship* (e.g., the NRPS-like BGC module). Together, these findings support the assertion that *Starship* activity helps generate selectable variation in BGC genotypes. The implication of this variation for the expression and diversification of natural products warrants further investigation.

### *Starships* encode clinically relevant phenotypes and are enriched in clinical strains

*Starships* encode genes with diverse functions typically associated with fungal fitness, including carbohydrate active enzymes, BGCs, and metal detoxification genes (Table 1, Table S5). To investigate broad trends in how *Starships* might contribute to phenotypic heterogeneity, we compared the predicted cargo functions among the 20 high-confidence *Starships* (Table S18; high-confidence data set; Materials and Methods). As expected, *Starship* types differ from each other in their predicted functional content, but functional diversity also varies to some extent within elements from the same type, indicating that both inter- and intra-specific *Starship* variations may contribute to functional heterogeneity among *A. fumigatus* strains. In this sense, different copies of the “same” *Starship* in different individuals have much greater potential to differ from each other at the sequence level compared with much smaller transposons. We found that 49.7% of cluster of orthologous groups (COG)-annotated genes in the high-confidence *Starships* are “Poorly Categorized”; 28.8% belong to the “Metabolism” category; 14.7% belong to “Cellular Processes and Signaling;” and 6.8% belong to “Information Storage and Processing.”

Given the importance of metabolic processes for diverse fungal traits, we examined granular classifications within the Metabolism category and found that *Starship* types differ specifically in their contributions to metabolic heterogeneity (Fig. S9). For example, nearly all 75 copies of *Gnosis h1* carry at least one gene with a predicted role in “Coenzyme transport and metabolism,” and none carry genes with predicted roles in “Lipid transport and metabolism,” while the exact opposite is true for the 119 copies of *Tardis h1*. The most frequently mobilized COG metabolism categories are “Carbohydrate transport and metabolism” (present in 371 individual elements belonging to 13 high-confidence *Starships*), “Secondary metabolite transport and metabolism” (present in 329 elements belonging to 16 high-confidence *Starship* types), and “Energy production and conversion” (present in 281 elements belonging to nine high-confidence *Starship* types), each of which has known associations with clinically relevant pathogen phenotypes. We found no evidence that any metabolism-associated COG category was enriched in *Starships* compared with background frequencies in Af293, indicating it is likely the specific mobilized functions, as opposed to an enrichment of certain classes of functions, that define *Starship* contributions to fungal phenotypes (data not shown).

While investigating associations between *Starships* and characterized pathogen phenotypes, we found three notable examples of cargo genes that encode known traits important for pathogen survival and virulence (Table S19; Materials and Methods) (10, 11, 37, 38, 40–45). The BGC encoding fumihopaside A (AFUA_5G00100-AFUA_5G00135) is carried by *Starship Gnosis h2* (Fig. 4A). Fumihopaside A is a triterpenoid glycoside that increases fungal spore survival under heat and UV stress exposure (37). Similarly, the BGC encoding the polyketide fumigermin (AFUA_1G00970-AFUA_1G01010), which inhibits bacterial spore germination, is carried by *Lamia h2* and *h4* (Fig. 4B) (36). Both the fumihopaside A and fumigermin BGCs were previously identified as “mobile gene clusters” based on their presence/absence at different chromosomal locations in *A. fumigatus* strains, and our results reveal that *Starships* are the mechanism underpinning their mobility (clusters 1 and 33, respectively, from reference 38).

Finally, a cluster of three genes (AFUA_5G14900/*hrmA*, AFUA_5G14910/*cgnA*, AFUA_5G14915/*bafA*) collectively referred to as the *hrmA*-associated cluster or HAC carried by *Nebuchadnezzar h1* increases virulence and low oxygen growth, and its expression modulates colony level morphological changes associated with biofilm development (Fig. 4D) (33, 46). The genes *bafB* and *bafC*, which are homologs of *bafA*, also mediate colony and submerged biofilm morphology and are each carried by up to five additional *Starships*, indicating a sustained association between this gene family and *Starships* (Fig. 4D, Fig. S10) (33). Mobilization of biosynthetic gene clusters and biofilm-related loci by *Starships* has the potential to contribute to the rapid evolution of these traits. For example, *Starships* are known to mediate rapid gene gain and loss through horizontal *Starship* transfer, which has recently been experimentally demonstrated using *A. fumigatus* as a recipient (28), and *Starship* loss, which has been experimentally demonstrated to occur through failed re-integration events (21). Thus, it is reasonable to predict that *Starship*-mediated gain and loss effectively increase the capacity of BGC and biofilm-associated traits to rapidly increase or decrease in frequency within populations; however, this hypothesis remains to be tested experimentally. Minimally, we propose that by mobilizing genes contributing to biofilm formation, *Starships* help drive heterogeneity in clinically relevant phenotypes at a population level (33).

To identify *Starships* that could be relevant in either environmental or clinical settings, we tested for an association between the high-confidence *Starships* and strain isolation source (genotyping data set; Materials and Methods). We found that five high-confidence *Starships* are significantly enriched (*P*_adj_ < 0.05) in strains from either clinical or environmental isolation sources across the 475/479 genotyped strains for which source data exist (Tables S2, S20, and S21; Fig. S11). *Lamia h3* (*P*_adj_ = 0.013) and *Janus h3* (*P*_adj_ = 0.034) are enriched in environmental strains. *Osiris h4* (*P*_adj_ = 0.022), *Nebuchadnezzar h1* (*P*_adj_ = 0.015; carries *bafA*; see above), and *Janus h2* (*P*_adj_ = 0.013) are enriched in clinical strains, providing complementary evidence to recently published analyses of *Starship* captain enrichment in clinical isolates across several other fungal species (47). Although clinical and environmental strains were sampled in roughly equal proportions, we cannot completely rule out biases in our sampling (e.g., phylogenetic and ecological biases) that would contribute to these enrichment patterns. Nevertheless, for each of the five enriched *Starships*, strains were often isolated from different countries (between three and 10 countries) and often belong to different phylogenetic groups (between one to three clades *sensu* [10] and one to six phylogenetic clusters *sensu* [11]), suggesting these *Starships* are excellent candidates to experimentally test how giant transposons contribute to fitness in environmental and clinical settings.

### *Starship* expression contributes to heterogeneity in a strain-, treatment-, and strain by treatment-dependent manner

The annotation of *A. fumigatus*’ *Starship* compartment next allowed us to test the hypothesis that *Starship* cargo is expressed under clinically relevant conditions. We analyzed patterns of differential *Starship* cargo gene expression in 14 publicly available transcriptomic studies from three commonly used reference strains (Af293, CEA10, A1163). In total, 177 transcriptome samples were included in this meta-analysis. Samples were split into broad treatment categories and corrected for batch effects to allow for comparison of cargo gene expressions between studies (Materials and Methods). Out of 596 *Starship* genes in total (carried by the high-confidence *Starships* present in the three reference strains) and across all treatments, we identified 459 DEGs that included both captain tyrosine recombinases and cargo (Supplemental material).

**Fig 5:**
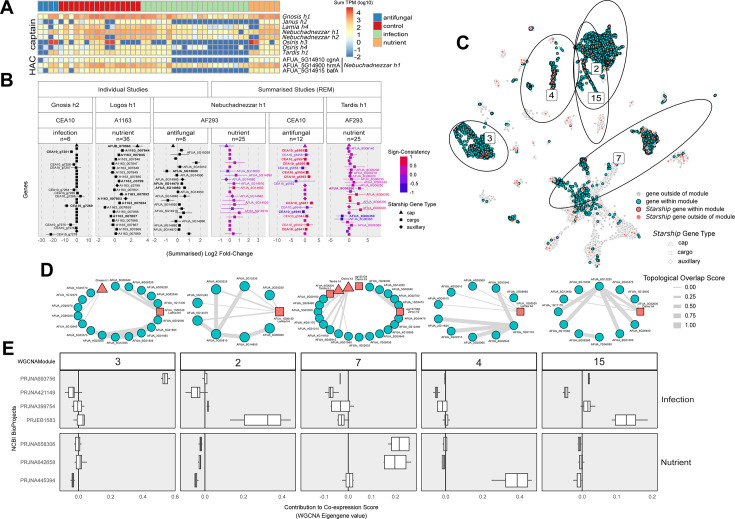
(A) A heatmap of transcript abundances (log_10_TPM) of *Starship* captain tyrosine recombinase genes (top) and genes within the *hrmA*-associated cluster (HAC; bottom) collapsed across treatment replicates for 11 RNAseq studies from *A. fumigatus* Af293. (B) Results from differential expression tests for select *Starships*, treatment categories, and strain combinations. Differentially expressed genes (DEGs) based on a single study are shown as log_2_ fold change (log_2_FC) in black with standard error bars, whereas DEGs identified across multiple studies are represented with summarized log_2_FC values from a random-effects model and colored by “sign-consistency” the number of studies that reported DEGs in the positive (+1) or negative (−1) direction centered around 0. Labels in bold font represent DEGs that are significantly differentially expressed in more than one study. (C) The results of a weighted gene co-expression network analysis (WGCNA) constructed from 14 “antifungal,” “infection,” and “nutrient” RNAseq studies represented using UMAP clustering (48) based on the co-expression eigengene values for all genes in the *A. fumigatus* Af293 genome. Non-*Starship* genes present within modules of interest are shown in blue, while all *Starship* genes are shown in red, with those genes within modules of interest having a black outline. (D) Genes within modules that were significantly associated with samples from specific treatment categories or studies were used to construct sub-networks, which contained the top 10 edges in the network that were made between any pair of genes or any gene and a *Starship* captain. The connections between genes (edges) are based on the topological overlap matrix for each module and have been 0–1 scaled. (E) Boxplots of eigengene values from WGCNA, akin to a weighted average expression profile, indicate the extent of co-expression (correlation) of the genes present within each module. Pairwise comparisons of module eigengene values determined if a module was significantly associated with samples from specific treatment categories or studies (Fig. S16).

To identify experimental conditions that may impact *Starship* transposition and the subsequent generation of *Starship*-mediated heterogeneity, we first examined patterns of transcript abundance for the captain genes of the 12 high-confidence *Starships* in Af293, CEA10, and A1163 (Fig. 5A, Fig. S12). All captain genes have evidence for constitutive gene expression (a minimum median transcript coverage of one across the gene body for biological replicates) in at least one experimental condition (Fig. S13). Overall, eight of the 12 captain genes from high-confidence *Starships* are differentially expressed in at least one study for one or more reference strains, revealing many opportunities for *Starship* transposition under lab conditions (Supplemental material). Differences in experimental conditions and/or strain backgrounds have no consistent positive or negative influence on captain gene expression, indicating that the intricacies of captain expression remain to be elucidated.

In order to identify conditions where *Starships* have the potential to impact phenotypes, we next examined patterns of differential expression for *Starship-*mobilized cargo genes. Overall, we did not find a significant enrichment of DEGs within *Starships* compared to the genomic background of any strain (Fisher’s exact test *P*-values > 0.05). However, the expression and significance of *Starship* DEGs vary across *Starship* naves, fungal strains, and experimental conditions, indicative of pervasive strain-specific, treatment-specific, and strain by treatment interactions impacting *Starship* cargo expression (Fig. 5B, Fig. S14). For example, we compared transcript abundance patterns for genes within the *hrmA*- and *baf-*associated clusters (46) between treatment categories and found *cgnA* and *bafA* to be down-regulated in the majority of samples assigned to infection treatments but not in control or nutrient treatment samples (Fig. 5A, Fig. S12). Together, fungal strain identity, treatment condition, and non-additive interactions between fungal strains and treatments generate variation in *Starship* cargo expression.

To gain further insight into *Starship*-mediated heterogeneity in gene expression and regulation, we constructed WGCNA, which visualizes correlations in gene expression. Genes are grouped into modules based on their level of shared co-expression patterns across all samples (Materials and Methods). We found five modules being significantly associated with “antifungal-,” “infection-,” or “nutrient”-based studies (Fig. 5E). We identified two major trends in the transcriptional network properties of *Starship* cargo. First, multiple *Starship*-associated genes are integrated into modules containing many other genes from the genome, underscoring the possibility that phenotypes emerging from these networks are the product of regulatory interactions between *Starship* cargo and non-*Starship* genes (Fig. 5D, Fig. S15). In particular, connections involving captain genes hint at candidate loci involved in regulatory interactions between *Starships* and the *A. fumigatus* genome and are prime targets for future investigations into the regulation of *Starship* transposition (Fig. 5E). Second, we also identified modules composed of mainly *Starship* cargo, indicative of modular networks specific to particular *Starships* (Table S24). Together, our network analyses paint a nuanced picture of how *Starships* interact with the broader regulatory networks of the cell and implicate them in the generation of transcriptional variation.

### Relevance and outlook

**Fig 6:**
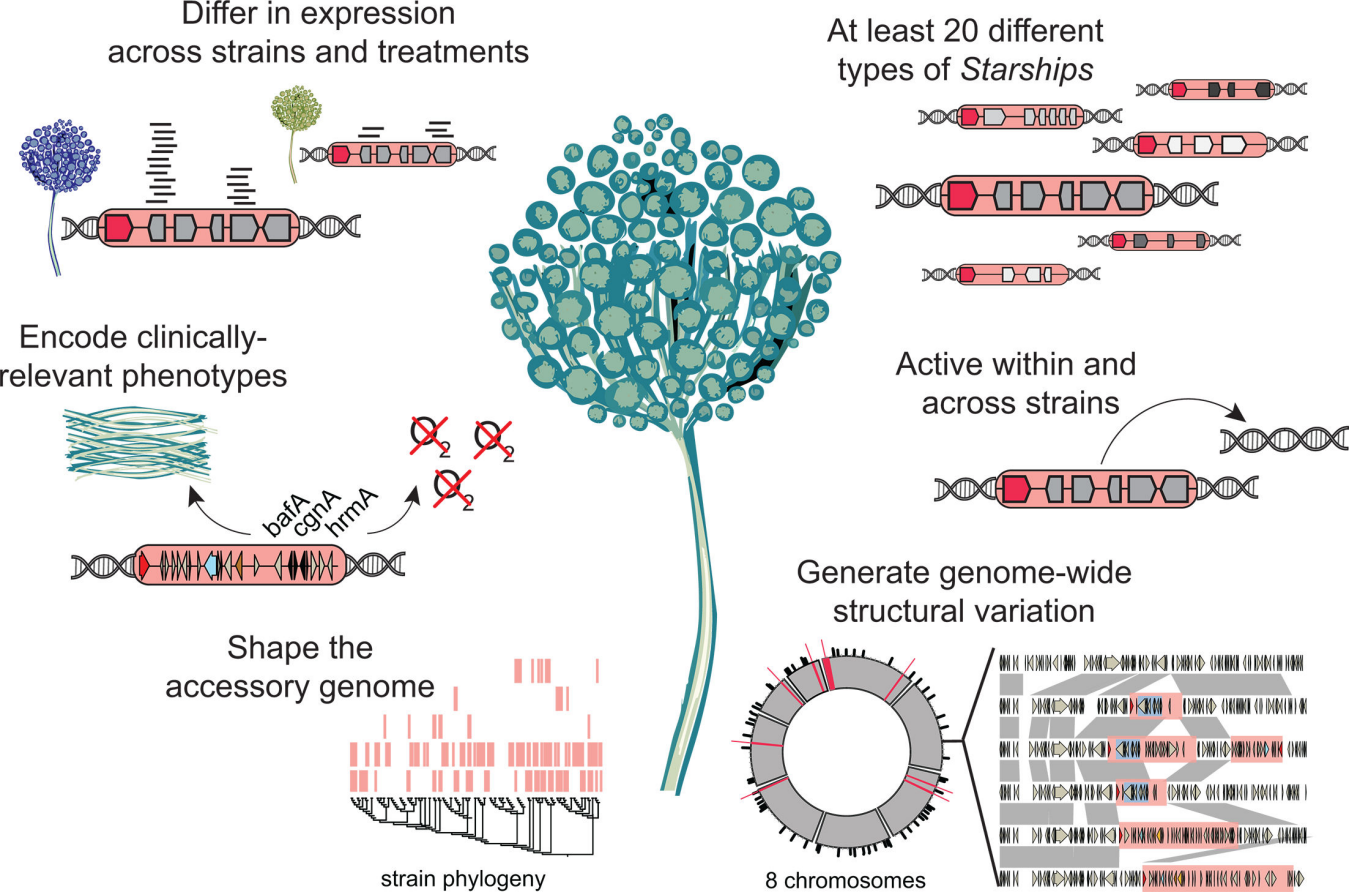
Contributions of *Starships* to strain heterogeneity in *Aspergillus fumigatus*.

Phenotypic heterogeneity among fungal pathogen strains poses a major challenge for combating infectious diseases, yet we often know little about the genetic basis of this variation. We hypothesized that a newly discovered group of unusual transposons, the *Starships*, makes important contributions to strain heterogeneity. We tested this hypothesis by systematically characterizing *Starship* presence/absence and expression in *A. fumigatus*, an important human fungal pathogen of critically high research priority (1, 49). Our work provides fundamental insight into the mode and tempo of fungal evolution by revealing that strain variation emerges not only from well-studied genetic mechanisms (e.g., single-nucleotide polymorphisms, copy number variation, chromosomal aneuploidies, and other types of structural variants) (10, 11) but from the previously hidden activity of giant *Starship* transposons (Fig. 6).

Although much remains unknown about the fundamentals of *Starship* biology, *Starships* in *A. fumigatus* likely generate variation on a scale approaching MGE-mediated evolution in bacteria, which is a major mechanism driving bacterial adaptation. The median number of *Starship*-borne genes per *A. fumigatus* isolate is 0.8% (range = 0–2.1%), while between 2.9 and 4.8% of the pangenome (corresponding to between 9.7 and 16% of the accessory genome) is *Starship*-associated. These observations approach analogous measurements of bacterial plasmids in the Enterobacteriaceae, where the median number of plasmid-borne genes per genome is estimated at 3.3% (range = 0–16.5%), and where between 12.3 and 21.5% of the pangenome is plasmid-associated (50). Given our requirements that a high-confidence *Starship* be found in two different locations in two different strains, our estimates represent a relatively conservative assessment of the mobile fraction of *A. fumigatus*’ genome. Mobility underpins prokaryotic genome dynamics, yet current models of fungal pathogen biology and evolution do not take gene mobility through transposition and horizontal transfer into account. These data highlight the need to understand the population-level impacts of *Starships* across different species and to integrate these findings into a new predictive framework for fungal biology. Our *Starship* compendium thus provides practical insights for elucidating *A. fumigatus* biology and establishes a roadmap for deciphering the origins of strain heterogeneity across the fungal tree of life.

## MATERIALS AND METHODS

### Long-read genome sequencing, assembly, and annotation

For DNA extraction, strains were grown at 37°C for 24 h in shaking liquid cultures using 1% glucose minimal media (51). Biomass was collected by gravity filtration through Miracloth (Millipore). High-molecular weight DNA extraction followed the fungal cetyltrimethylammonium bromide (CTAB) DNA extraction protocol (52). Biomass was ground in liquid nitrogen and incubated at 65°C in a lysis buffer composed of 650 µL of buffer A (0.35 M sorbitol, 0.1 M Tris-HCl [pH 9], 5 mM EDTA [pH 8]), 650 µL buffer B (0.2 M Tris-HCl [pH 9], 0.05 M EDTA [pH 8], 2M NaCl, 2% CTAB), 260 µL of buffer C (5% Sarkosyl), 0.01% PVP, and 0.2 mg proteinase K for 30 min. Potassium acetate (280 µL of 5 M solution) was added and incubated on ice for 5 min. DNA was extracted by phenol:chloroform:isoamyl alcohol extraction, followed by a secondary extraction with chloroform:isoamyl alcohol. The supernatant was RNase-treated (2.5 µg) and precipitated using sodium acetate (0.3 M) and isopropanol. DNA was finally purified using 70% ethanol, dried, and rehydrated in TE (pH 9). To preserve long strands of DNA suitable for long-read sequencing, samples were gently mixed by inversion and transferred using large bore pipette tips. DNA quality was assessed using NanoDrop and concentration quantified using Qubit dsDNA BR Assay Kit. Finally, DNA fragment size was assessed on 1% agarose gel, looking for large quantities of DNA to remain in the well after 1 h of running at 100 v. DNA was sent to SeqCoast for Oxford Nanopore Sequencing. We used previously sequenced Illumina short-read data for polishing the assemblies with Pilon (10). Two strains not previously sequenced were sequenced with Illumina at SeqCoast.

The long-read genome assemblies were assembled with Canu v2.2 and polished with five rounds of Pilon v1.24 wrapped with AAFTF v0.3.0 (53–55). Summary statistics for each assembly were computed with AAFTF and BUSCO v5.7 using eurotiomycetes_odb10 marker set (56). Genome annotations (including for the publicly available CEA10 assembly) were performed using Funannotate v1.8.10, which trained gene predictors with PASA using RNA-Seq generated for each strain (Puerner et al., forthcoming), and predicted genes *de novo* in each assembly and produced a consensus gene prediction set for each strain (57, 58).

### Functional predictions

We annotated all predicted genes with Pfam domains, InterPro domains, and Gene Ontology (GO) terms using InterproScan v5.61-93.0 (-appl Pfam --iprlookup --goterms) (59). We annotated carbohydrate active enzymes using dbcan v3.0.4 (--tools all) and EggNOG orthogroups and COG categories using eggnog-mapper v2.1.7 (--sensmode very-sensitive --tax_scope Fungi) (60, 61). We annotated biosynthetic gene clusters using antiSMASH v6.0.1 (--taxon fungi --clusterhmmer --tigrfam) and 5S rDNA using infernal v1.1.4 (--rfam -E 0.001) (62, 63). On average, 35.7% of genes within *Starships* have no known PFAM or InterPro domain, GO term, COG category, or eggNOG orthology (range = 0–67.6%, SD = 14.8%), but approximately half of all mobilized genes could be assigned to a COG (e.g., 50% of 11,900 genes total, across 459 high-confidence elements). To investigate associations between *Starships* and pathogen phenotypes, we searched for the presence of 798 genes known to be associated with virulence or infection-relevant phenotypes on *Starships* (Table S19) (10, 11, 37, 38, 40–45).

### Single-nucleotide polymorphism analysis

We calculated pairwise kinship among strains by analyzing a previously generated variant call format of all high-quality, filtered, biallelic single-nucleotide polymorphisms (SNPs) from the Lofgren et al. population with plink v1.9 (--distance square ibs flat-missing --double-id --allow-extra-chr; Fig. S3) (64, 65). We calculated Jaccard similarity in *Starship* repertoire among all pairwise combinations of strains using genotyping data for the set of 20 high-confidence *Starships* and the following formula: *Starships* shared between strains 1 and 2 / (*Starships* unique to strain 1 + *Starships* unique to strain 2 + *Starships* shared between strains 1 and 2).

### Pangenome construction

We constructed a pangenome for the 13 reference-quality strains (our 11 long-read assemblies plus the Af293 and CEA10 references) combined with the 506 publicly available short-read assemblies (10, 11) using Orthofinder v2.5.4 (--only-groups -S diamond), and then extracted all orthogroups with a sequence from at least one of the 13 reference-quality strains (Fig. 2B) (66). Core orthologs are defined as being present in all 13 strains; accessory orthologs are present in between two and 12 strains; and singleton orthologs are present in one isolate. We included all 519 strains in the Orthofinder analysis to ensure that ortholog groups are consistent across different population subsets.

### BLAST analysis

We determined the genomic locations of all best reciprocal BLAST hits to cargo genes carried by the 20 high-confidence *Starships* using BLASTp v2.13 (Fig. 1C) (67). For each cargo gene, we retrieved the highest scoring hit with ≥90% identity and ≥90% query coverage in each of the 13 reference-quality assemblies and determined whether that hit was found in the same *Starship*, a different *Starship*, or not in a *Starship* at all. If no hit was retrieved, that gene was marked as missing.

### Within strain analysis

To assess *Starship* transposition among isolates of the same strain, we compared six independently sequenced data sets of CEA10 and CEA10-derived strains to the long-read CEA10 reference assembly. Genome codes and National Center for Biotechnology Information (NCBI) accessions are as follows: CEA10-1: SRR7418934, CEA10-2: ERR232423, CEA10-MCH: ERR232426, A1160: SAMN28487500, A1163: SRR068950, A1163-Cramer_SM: SRX27799936. We evaluated three additional strains that also met the criteria of having a reference quality long-read assembly and at least one independently generated sequencing data set generated by other research groups: ATCC46645, S02-30 (=AF100-9B), and TP9 (=08-19-02-30). Short-read sequence data were mapped to our long-read assemblies with bwa-mem2 and visually inspected in IGV to determine if any *Starships* were deleted/transposed (68). Short-read data from select isolates with evidence of transposition were *de novo* assembled with SPAdes, and existing gene annotations were mapped from long-read assemblies using liftoff (69, 70).

### *Starship* annotation

We systematically annotated *Starships* in the 12 newly sequenced long-read genomes plus 507 publicly available *A. fumigatus* genomes by applying the starfish workflow v1.0.0 (default settings) in conjunction with metaeuk v14.7e284, Mummer v4.0, CNEFinder, and BLASTn (Table S2) (22, 67, 71–73). Briefly, captain tyrosine recombinase genes were *de novo* predicted with starfish’s Gene Finder Module, and full-length elements associated with captains were predicted with starfish’s Element Finder Module using pairwise BLAST alignments to find empty and occupied insertion sites. We filtered out all elements that were <15 kb in length, which we have found to correspond to indels of captain genes but never to the transposition of a full-length *Starship* (22). We then manually examined alignments between each putative *Starship* and its corresponding insertion site and filtered out all “low confidence” poorly supported alignments indicative of a false positive insertion. Poor alignments were observed most often when *Starships* were inserted into smaller transposons, when an inversion breakpoint occurred within a putative *Starship*, resulting in an overestimation of *Starship* length, or when the insertion site was on a very small contig, resulting in small flanking region alignments.

We verified and supplemented these automated *Starship* predictions with manual annotations of the three reference strains Af293, CEA10, and A1163, along with a set of 86 additional elements (Tables S3 and S7). Insertion sites and DRs were manually verified by generating alignments of the elements plus the 50 kb flanks to a corresponding putative insertion site, as determined by starfish (Table S4). MAFFT v7.4 was used to generate alignments with default parameters (74). The DRs were visually assessed, and the insertion regions were examined for the presence of TEs, which could lead to erroneous target site, DR, and TIR determinations. For *Starships* in the three reference genomes, the set of reference *Starships* was used as a query with BLAST to verify their start and end coordinates and insertion sites. Additionally, the starfish output was examined for additional elements, which did not meet our initial strict cutoffs to attempt to capture the entire *Starship* repertoire of these strains.

### *Starship* classification

*Starships* were then grouped into naves (singular: navis, latin for “ship”) by clustering captain sequences in ortholog groups using mmseqs easy-cluster (--min-seq-id 0.5c 0.25 --alignment-mode 3 --cov-mode 0 --cluster-reassign) (75). *Starship* sequences were then grouped into haplotypes using the MCL clustering algorithm in conjunction with sourmash sketch (-p k = 510 scaled = 100 noabund) that calculated pairwise k-mer similarities over the entire sequence length, as implemented in the commands “starfish sim” and “starfish group” (76, 77). These automated and systematic predictions yielded a core set of 787 individual elements grouped into 54 distinct navis-haplotype combinations.

We identified segregating insertion sites associated with the 787 elements across all 519 strains using the command starfish dereplicate (--restrict --flanking 6 --mismatching 2) in conjunction with the Orthofinder orthogroups file (see above) that was filtered to contain groups absent in at most 517 strains and present in at most eight copies per isolate. Starfish dereplicate enables the identification of independently segregating insertions by grouping *Starships* and their insertion sites into homologous genomic regions using conserved combinations of orthogroups between individuals. We genotyped the presence of each navis-haplotype combination within each region for each isolate. If an isolate did not have any *Starships* within a given region, it was assigned either an “empty” genotype (if the set of orthogroups that defines the upstream region flank was adjacent to the set of orthogroups that defines the downstream region flank) or a “fragmented” genotype (if additional orthogroups were in between the upstream and downstream region flanks).

To define high-confidence *Starships*, we used a threshold that required observing the same navis-haplotype in ≥2 independent segregating sites in different strains, which ensured an accurate and precise determination of each element’s boundaries and the sequences therein (22). We found 18 navis-haplotype combinations meeting these criteria. We considered two additional navis-haplotype combinations of interest (*Osiris h4* and *Lamia h4*) to be high-confidence after manually annotating their boundaries and finding evidence for flanking direct repeats that are signatures of *Starship* boundaries. Each high-confidence *Starship* is represented by a type element (similar in concept to a type specimen for defining a biological species) that constitutes the longest element assigned to that navis-haplotype. The type elements of the reference *Starships* range in size from 16,357 to 443,866 bp and collectively represent 459 individual elements with an average length of 71,515 bp (Tables S4 and S5). The remaining 328 elements have an average length of 50,249 bp and are represented by 34 “medium-confidence” navis-haplotypes that were only observed at a single segregating site. We predicted the target sites of each high-confidence *Starship* by manually aligning multiple empty insertion site sequences to each other and identifying columns within 25 bp of the core motif that had conserved nucleotides in at least 80% of sequences (Table 1).

Each navis associated with a high-confidence element was assigned a charismatic name (e.g., *Tardis*) and a new, sequentially named haplotype (*h1*, *h2*, etc.) to distinguish it from the automatically generated haplotype codes (*var14*, *var09*, etc.). All other 34 navis*-*haplotype combinations with evidence of only a single segregating site were classified as “medium-confidence.” Medium-confidence *Starships* with captains not belonging to the high-confidence naves kept their automatically generated navis and haplotype codes (e.g., *navis01-var09*), while medium-confidence *Starships* with captains belonging to the high-confidence naves were assigned to that navis but kept their automatically assigned haplotype (e.g., *Osiris-var04*).

### *Starship* genotyping

Starfish requires a well-resolved empty insertion site in order to annotate the boundaries of a contiguous *Starship* element. It will, therefore, not annotate any element whose corresponding empty site is missing or any element that is partially assembled or not assembled at all. We, therefore, supplemented starfish output using a combination of BLASTn and short-read mapping to the library of reference *Starship* sequences to decrease the false negative rate for presence/absence genotyping. First, we recovered full-length and partial elements using the set of high-confidence *Starships* as input to the command “starfish extend.” This command uses BLASTn to align full-length *Starship* elements to the sequence downstream from all tyrosine recombinase genes not affiliated with a full-length *Starship* element.

Separately, we downloaded publicly available paired-end short read Illumina sequencing data for all 466 strains for which these data were available (10, 11) and mapped them to the library of 20 high-confidence *Starships* using the command “starfish coverage” and the aligner strobealign (78), ensuring that we would correctly genotype the presence/absence of a *Starship* even if it is not present in an assembly or partially assembled. By mapping short reads directly to a library of known *Starship* elements, we overcame some of the drawbacks associated with working with short-read assemblies. We considered any *Starship* with a minimum of five mapped reads at each position across 95% of its entire length to be present. We considered a *Starship* to be present in a given individual if either the main starfish workflow, BLASTn extension, or short-read mapping identified it as present; otherwise, we considered it absent.

### Meta-analysis of *A. fumigatus* RNAseq data sets

We collected transcriptomes from a total of 434 publicly available paired-end libraries of *A. fumigatus* strains Af293, A1163, and CEA10/CEA17 (Table S22). We surveyed the available metadata from each BioProject and binned the samples into general categories based on the type of treatment applied in each study. The categories that were the most well represented across multiple BioProjects include exposure to antifungals (“antifungal”), *in vivo* and *in vitro* infection experiments (“infection”), or supplemented growth media with specific nutrients (“nutrient”; Tables S22 and S23).

RNAseq reads were retrieved from the NCBI SRA using fasterq-dump from the SRA toolkit (last accessed 26 June 2024: https://github.com/ncbi/sra-tools) and trimmed for quality and sequencing artifacts using TrimGalore (last accessed 26 June 2024: https://github.com/FelixKrueger/TrimGalore). Transcripts were quantified using Salmon quant, which employs a reference-free pseudo-mapping-based approach to quantify transcripts. In addition to the pseudo-BAM files created by Salmon v1.10.1 (79), we created separate sets of BAM files to assess transcript coverage across *Starship* genes by mapping to the transcriptome with STAR v2.7.11a (80). We assessed transcript coverage across the core of *Starship* genes using the bamsignals R package (last accessed 26June 2024: https://github.com/lamortenera/bamsignals). Genes with a median core-transcript coverage less than one were considered to have insufficient evidence of being expressed. We applied an abundance filter on transcript abundances, removing genes represented by fewer than 10 transcripts in at least three samples. We corrected for batch effects between BioProjects using CombatSeq (81) and excluded BioProjects with inconsistent/unclear metadata or those that remained as outliers in the principal component analysis after batch correction. DESeq2 v1.45.0 (82) was used to perform a series of differential expression tests using the corrected transcript counts. These tests were performed individually for each BioProject, with each test comparing the differences in expression between all control and treatment samples. Differences in treatment level were included as a cofactor in the model, where applicable.

To summarize expression patterns across BioProjects, which tested similar conditions, we employed a random effects model (REM) using the R package metaVolcanoR (83). This method identifies differentially expressed genes (DEGs) as genes that are significantly differentially expressed in all studies and account for the variation in the expression for each DEG observed in multiple BioProjects. The output from the meta-volcano REM includes summarized log2 fold-change, a summary *P*-value, and confidence intervals for each DEG. In addition, a measure of “sign-consistency” is used to evaluate the consistency of DEG, which is expressed as a count of the number of BioProjects where a DEG was observed with the same directional change (+/−) centered around 0.

We performed weighted gene co-expression network analysis (WGCNA) using PyWGCNA v1.72-5 (84) to construct gene co-expression networks for *A. fumigatus* reference strain Af293. A single network was constructed using the batch-corrected transcripts per million values from the collection of samples belonging to “antifungal,” “infection,” and “nutrient” treatment categories. PyWGCNA automatically estimates an appropriate soft-power threshold based on the lowest power for fitting scale-free topology and identifies modules of co-expressed genes through hierarchical clustering of the network and performing a dynamic tree cut based on 99% of the dendrogram height range. The topological overlap matrix is then computed, and a correlation matrix is constructed to produce the final network. Distributions of topological overlap matrix (TOM) scores were generated based on edges in the network between genes within *Starships*, between *Starships*, between *Starships* and the rest of the genome, and between non-*Starship* genes in the genome. We compared these distributions using a Wilcoxon test and analysis of variance, adjusting *P*-values with the Holm-Bonferroni method. Modules in the network that were significantly associated with samples from a specific treatment category were identified using the pairwise distances between observations using pdist from the Python module SciPy (85). To highlight the genes within each module that have the most strongly correlated expression profiles, sub-networks were constructed using only the edges with the 10 highest TOM scores made between either *Starship* captain genes or any non-*Starship* gene that was present in the module. The collections of genes within these modules were also tested for functional enrichment using gprofiler2 (86).

### Statistical tests, alignments, and data visualization

Enrichment tests were performed with either the Binomial test (binom.test; alternative = “greater”) or the Fisher’s exact test (fisher.test; alternative = “greater”) implemented in base R using the p.adjust function (method = Benjamini-Hochberg) to correct for multiple comparisons. Mantel tests for matrix correlations were performed in R (method = "pearson", permutations = 999). We conducted nucleotide alignments among *Starships* and their associated genomic regions (Fig. 1E and 3D) using Mummer 4.0 with the nucmer command with delta-filter options -m -l 5000 -i 95 (71). We visualized all alignments and insertion site distributions using Circos and gggenomes (87, 88) with the help of wrapper script functions implemented in the function starfish locus-viz in the starfish package v1.1 (22). Step-by-step tutorials for *Starship* data visualization are maintained on the starfish github repository available at https://github.com/egluckthaler/starfish/wiki. All other figures were generated in R using ggplot2 (89).

## Data Availability

Sequencing reads and genomic assemblies for the 11 isolates sequenced with Oxford Nanopore have been deposited and accessioned at NCBI (Table S1). Short-read sequence data for the A1163-Cramer_SM isolate are deposited at NCBI under Bioproject PRJNA1228067. We downloaded the Af293 and CEA10 reference assemblies (NCBI accessions: GCF_000002655.1 and SAMN28487501), as well as 252 assemblies and annotations from Barber et al. (11) from NCBI and 256 assemblies and annotations from Lofgren et al. from the associated Zenodo repository (64). We subsequently reformatted the contig and gene identifiers to include genome codes generated as part of this study (Table S2). All scripts, including raw data for figure generation, genomic data used for this study, and results from differential expression tests are available through the following Figshare repository: DOI: 10.6084/m9.figshare.26049703.

## SUPPLEMENTAL MATERIAL

Supplemental material mbio.01092-25-s0001.pdf Supplemental results, figures, and table captions.

Supplemental tables mbio.01092-25-s0002.xlsx Tables S1 to S25.

ASM does not own the copyrights to Supplemental Material that may be linked to, or accessed through, an article. The authors have granted ASM a non-exclusive, world-wide license to publish the Supplemental Material files. Please contact the corresponding author directly for reuse.
