# Supplementary material for: Giant transposons promote strain heterogeneity in a major fungal pathogen
Source: mBio. 2025 May 12;16(6):e01092-25. doi: 10.1128/mbio.01092-25 (PMC12153272; doi:10.1128/mbio.01092-25)
Supplement: Supplemental material — Supplemental results, figures, and table captions. [file mbio.01092-25-s0001.pdf]

# Supplemental Material

## *Starship* frequencies

The population-level frequency of high-confidence *Starships* ranges from 1-60%, with 13 of the high-confidence *Starships* present in <10% of strains and no *Starship* present in more than 60% of strains (Table S6). Each high-confidence *Starship* is present across multiple individuals; however, any given *Starship* element is only present at most once in a genome, with only 3 exceptions that likely involve recently degraded copies, suggesting some mechanism for controlling their copy number (e.g., repeat-induced point mutation (21)). Individual elements typically segregate at low frequencies, with each segregating site containing on average an inserted element in just 0.4% of strains across the 20 high-confidence *Starships* (range = 0.2-52.3%, std. dev. = 6.5%, n = 154; Fig. 3B). However, multiple insertion sites often exist for a given element such that each high-confidence *Starship* is present on average in 4.35 different regions (range = 1-15, std. dev. = 3.92).

## Differential expression of *Starship* captains and cargo

Patterns of transcript coverage, transcript abundance, and differential expression of *Starship*-associated genes were generally heterogeneous, with various strain-, treatment- and strain by treatment interactions. The transcriptional profile for many *Starships* is patchy, and not all *Starship*-mobilized genes are expressed in response to experimental conditions. Only certain cargo genes have the minimum required evidence for constitutive expression: a median transcript coverage of 1 across the gene body. Nearly all genes in *A. fumigatus* CEA10 *Starships* surpass this threshold (Fig. S13B, across all samples from the various studies we included in our analyses. One notable exception being the captain gene in *Janus h2*, which is sparsely expressed across most studies. In comparison, genes present in *Starships* from Af293 (Fig. S13A) and A1163 (Fig. S13C) have fewer genes that surpass this threshold, with certain experimental conditions having more consistent expression of the *Starship* genes in these strains. For example, specific studies conducted on Af293 or CEA10, testing exposure to heatshock (PRJDB6203), hypoxia (PRJNA144647), *in vitro* infections (PRJEB1583, PRJNA399754, PRJNA399754). and various gene knock-out experiments (PRJNA390719, PRJNA396210, and PRJNA601094) have consistently reported expression of all genes across the *Starship* element.

Captain genes of multiple *Starships* from Af293 and CEA10 had low transcript abundance within "infection" studies. Furthermore, samples of a study performing an experimental infection using a murine model (PRJNA421149) had near-zero transcript abundances of captain genes from Af293 *Starships Janus h2*, *Osiris h3*, and *Gnosis h1*. However, this was not found within samples of a different study of an Af293 infection of human pneumocyte cell lines (PRJNA399754). Near-zero abundances were also found for a different set of captain genes, within CEA10 *Starships Tardis h1, Logos h2,* and *Gnosis h2*, from an *in vitro* infection study using human cell lines (Fig. S12), suggesting that the transcriptional response of *Starship* captains within the infection environment differs between strains.

We used tests of differential expression using existing *A. fumigatus* RNAseq studies to identify which environments or conditions influence the expression of genes within the *Starship* compartment (Fig. 5B, Fig. S14, Table S23). In particular, the change in expression of *Starship* captain genes within studies that applied similar types of treatments may have implications for what conditions may be more conducive for transposition. In general, multiple Af293 RNAseq "infection" studies have a prevailing negative impact on the expression of captain genes. Starships *Lamia h4*, *Tardis h1*, and *Gnosis h1* all had significantly lower expression within captain genes compared to their respective control samples (Figure 5B). We also observed a negative impact on captain expression within "antifungal" studies, which consisted of multiple studies conducted on Af293 and CEA10 with exposure to the antifungal caspofungin. Treatments of caspofungin were found to decrease the expression of Starship captains within Af293 Nebuchadnezzar h1 and CEA10 *Logos h2*. However, caspofungin was also found to be associated with increased expression of Af293 *Tardis h1* and *Gnosis h1* captain genes. Specific "nutrient" studies were also found to have a positive influence on captain gene expression. This includes a single study exposing A1163 to glucose/xylose media (PRJNA634102), which significantly increased the expression of captain genes within *Logos h1* and *Nebuchadnezzar h1*, compared to the control group grown on minimal media. Together, the differential expression results across these gene expression datasets demonstrate the context-dependent expression of captain genes, which we speculate would lead to variation in *Starship* transposition rates across different environments and strain backgrounds.

Similar to captain genes, the expression patterns of cargo genes within *Starships* are heterogeneous. The transcriptional profile of *Starships* is punctuated with expression of cargo genes (Fig. 5B, Fig. S14, Table S23), which can have sufficient transcript coverage even in the absence of captain expression, suggesting that the transcriptional regulation of these cargo genes are decoupled from transposition, and could be integrated into the existing genomic transcriptional networks. Of particular note are the genes that form biosynthetic gene clusters (BGC) which reside within *Starships*. In Af293, 63 BGC genes across seven Starships (*Gnosis h1, Lamia h4, Nebuchadnezzar h1, Nebuchadnezzar h2, Osiris h4, Tardis h1*, and *nav10 var35*) are differentially expressed in one or more of the treatment categories investigated in this study. At least one gene from the BGCs across these seven *Starships* are differentially expressed in response to caspofungin exposure, from a specific Af293 study (PRJNA472460). Furthermore, multiple genes present in Osiris h4 which are associated with another BGC (AFUA_3G02580, AFUA_3G02620, AFUA_3G02630, AFUA_3G02640) have significantly increased expression in nutrient studies compared to controls. Interestingly, generally fewer genes in BGCs are differentially expressed in Af293 "nutrient" studies.

As an opportunistic human pathogen, we were also interested in *A. fumigatus* genes that have specific relevance to virulence, such as genes within the *hrmA*-associated cluster (HAC). Two HAC genes present in *Nebuchadnezzar h1*, *cgnA* and *bafA*, have near-zero transcript abundances within samples from an Af293 study of an experimental infection of a murine model (PRJNA421149). However, genes within the HAC cluster were only differentially expressed within certain CEA10 antifungal studies and a single infection study: we found *bafA* in *Nebuchadnezzar*

*h1* (CEA10_g5945) and *bafZ* in *Logos h1* (CEA10_g7202) to both have decreased expression in these studies, respectively (Fig. 5B, Fig. S14).

Certain cargo genes within Starships demonstrate patterns of differential expression in response to various experimental conditions, suggesting that these genes play a functional role in metabolic or enzymatic activity of the cell in response to specific stimuli. In Af293, we identified several Starship cargo genes with condition-specific expression patterns. Within *Nebuchadnezzar h1*, a proteoglycan 4 gene (AFUA_5G14930) showed increased expression during infection.

A group of cargo genes within Af293 *Lamia h4* displayed complex expression patterns across various treatment categories in this study. Within “infection” studies, multiple genes with diverse functions had decreased expression, including genes relevant to metabolic functions ( AFUA_1G00800; flavin-containing amine oxidoreductase, AFUA_1G00340; thioesterase, and AFUA_1G00490; dihydrolipoamide acetyltransferase component of pyruvate dehydrogenase complex). One gene relevant to post-transcriptional modification also had decreased expression (AFUA_1G00730; DNAj domain protein). Few genes within Lamia h4 that serve potential roles in redox processes had increased expression within “infection” or “nutrient” treatments (AFUA_1G00440; DUF895 domain membrane protein and AFUA_1G00350; FAD-binding domains, respectively).

In CEA10, several genes showed increased expression with “antifungal” treatments, including genes containing NAD(P)-binding (CEA10_g9263; *Janus h2*) and PAS superfamily protein (CEA10_g5959; *Nebuchadnezzar h1*), and phosphotyrosine protein phosphatase (CEA10_g5954; *Nebuchadnezzar h1*). While several cargo genes were consistently downregulated in CEA10 *Logos h2*, including those encoding WD40 repeat proteins (CEA10_g5566), P-loop containing nucleoside triphosphate hydrolase (CEA10_g5567), homeodomain-like superfamily proteins (CEA10_g5587), and serine/threonine and tyrosine kinase (CEA10_g5577).

In response to “nutrient” conditions, several cargo genes within A1163 *Logos h1* were differentially expressed. with increased expression of serine threonine-protein (A1163_007845), DUF3723, MADS-box transcription factor (A1163_007852), and freB (A1163_007853). Conversely, a BTB domain-containing gene (A1163_007857), potentially involved in transcriptional regulation through chromatin structure control, showed decreased expression under the same conditions.

Additional cargo genes of interest may convey resistance to environmental stressors, including genes that provide resistance to environmental toxins. Genes incorporated in the arsenic detoxification pathway are present within *A. fumigatus* Starships *Nebuchadnezzar h1* within strains Af293 and CEA10. Arsenite methyltransferase genes were found to have significantly decreased expression in the Af293 copy (AFUA_5G15000) and significantly increased expression in the CEA10 copy (CEA10_g5956) with exposure to caspofungin (Fig. 5B). Collectively, these findings highlight the diverse functional roles of Starship cargo genes in responding to environmental stressors and nutrient availability, suggesting their importance in adaptability and survival of *A. fumigatus*.

## *Starship* co-expression networks

We performed weighted gene co-expression network analysis (WGCNA) using *PyWGCNA* to construct gene co-expression networks for *A. fumigatus* reference strain Af293. Overall, we found that genes within or between *Starships* are more strongly co-expressed together than with non-*Starship* genes, based on pairwise comparisons of the distribution of scores in the topological overlap matrices (TOM) (corrected p-values < 0.01 for all comparisons; Fig. S15). This suggests that a tighter transcriptional relationship exists between genes mobilized by *Starships* in Af293 compared to co-expression with other genes in the genome.

Genes in the *A. fumigatus* Af293 co-expression network were resolved into 27 modules (Table S24). The majority of genes found within each module are not contained within a *Starship*, yet almost all modules contain one or more *Starship* genes, including those previously identified as DEGs (Table S24). Modules were selected for further analyses based on the correlation of their gene expression profiles (WGCNA eigengenes) and their association with a treatment category ("antifungal", "infection", or "nutrient") or specific study (Fig. 5E, Fig. S16).

To investigate functional compartmentalization of WGCNA modules, we performed enrichment tests and identified which GO/KEGG terms are significantly overrepresented within each module. Genes in module "2" are significantly enriched in functions including the production of antimicrobial secondary metabolites (fumagillin), the secretion of *A. fumigatus* mycotoxins (helvolic acid), proteins for heme binding, and synthesis of immunosuppressive compounds (endocrcoin) (Table S25).

To identify which genes are most strongly co-expressed with *Starship* cargo or captain genes, we subsetted the co-expression network to keep only the top 10 edges that were made between any pair of genes or any gene and a *Starship* captain. Two modules within the Af293 co-expression network ("2" and "15") are significantly more commonly co-expressed within samples from a single infection study of Af293 (PRJEB1583; Fig. 5E). The connections with the highest TOM scores in module "2" include those between an IBR finger domain protein within the *Starship Lamia h4* (AFUA_1G00150) and genes involved in RNA binding pathways (AFUA_6G12070), as well as genes for alpha-amylase (AFUA_2G03230) and anthrone oxygenase (AFUA_4G00225) (Fig. 5D). Genes within the module "15" include those tightly connected to the expression of F-box domain protein within *Lamia h4*. Genes within module "15" are enriched in glycerophospholipid metabolism (Table S25). These co-expression relationships provide insight into how *Starships*-mobilized genes integrate into the existing transcriptional network in *A. fumigatus* strains.

Module "3" is significantly associated with increased expression within a single study of an experimental infection in mice (PRJNA693756) (Fig. 5E, S16). Genes within module "3" are enriched for genes in ribosome biogenesis in eukaryotes, carbon metabolism, and pyruvate metabolism (Table S25). The connections with the highest TOM scores in module "3" include genes co-expressed with *Gnosis h1* captain gene: genes with predicted catalytic activity (AFUA_1G12370), zinc-containing alcohol dehydrogenase (AFUA_2G00970), mitochondrial respiration (AFUA_2G06020), and exonuclease activity/DNA-directed DNA polymerase activity/role in mitochondrial DNA replication and mitochondrion localization (AFUA_5G12640).

Module “7” is significantly associated with two studies testing supplementation with 5,8-dihydroxyoctadecadienoic acid (PRJNA658306) and lipo-chitooligosaccharides (PRJNA642658) in Af293 (Fig. 5E, S16). The focus of both of these studies is to understand how these supplementations impact the regulation of fungal growth and development. The connections with the highest TOM scores in the module “7”, include genes co-expressed with captain genes of *Tardis h1* and *Osiris h3. Osiris h3* captain co-expressed with predicted RNA binding, ribonuclease III activity and role in RNA processing (AFUA_3G03050). The connections made in this module with *Starship* genes present themselves as good candidates for future research to understand which genes are expressed along with transposition.

# Supplemental table legends

Table S1: Genome assembly statistics for the 12 strains sequenced with Oxford Nanopore long-read technology

Table S2: Metadata for publically available *Aspergillus fumigatus* strains

Table S3: Manual annotation data of 86 Starship elements

Table S4: Metadata for all starfish-predicted Starships

Table S5: Sequence features of all starfish-predicted Starships in the 519 strain population

Table S6: Frequencies of the 20 high-confidence Starships in the 519 strain population using the expanded dataset

Table S7: Manual annotation of Starship elements in three *Aspergillus fumigatus* reference strains

Table S8: Pairwise comparisons between strains of SNP identity by state and Starship repertoire similarity

Table S9: Comparison of Starship coordinates with structural variants identified by Colabardini et al 2022 (doi: 10.1371/journal.pgen.1010001)

Table S10: Orthogroup frequencies in the 519 strain population

Table S11: Orthogroup frequencies in the 13 reference-quality strains

Table S12: BLAST recovery of Starship cargo genes from the 13 reference-quality strains

Table S13: BLAST recovery of Starship cargo genes from the 519 strain population

Table S14: Genotyping of genomic regions with segregating Starship insertions

Table S15: Starships in genotyped genomic regions with segregating Starship insertions

Table S16: Summary of genotyping data for genomic regions with segregating Starship insertions

Table S17: Coordinates of predicted 5s rDNA sequences in the 519 strain population

Table S18: Proportion of elements carrying at least one gene annotated with various COG categories

Table S19: List of putative and published virulence and stress resistance genotypes in *Aspergillus fumigatus*

Table S20: Fisher's exact test statistics for Starship enrichment by strain isolation source

Table S21: Presence/absence genotyping data for the 20 high-confidence Starships in the 519 strain population using the genotyping dataset

Table S22: Metadata from RNASeq studies collected from NCBI used in the meta-analyses of *Starship* gene expression.

Table S23: Summary of RNASeq studies collected from NCBI used in the meta-analyses of *Starship* gene expression.

Table S24: Summary of modules assigned, and genes within them, from the WGCNA constructed from Af293 samples.

Table S25: Significantly enriched functional terms from a series of enrichment tests (Fisher's Exact Test) conducted on the genes within WGCNA modules.

# Supplemental figure legends

Figure S1: Counts of starfish-predicted *Starships* per *Aspergillus fumigatus* strain (n = 519), broken up by assembly project (either the 12 Oxford Nanopore Assemblies generated by study plus the AF293 reference genome, or from Lofgren et al 2022 or Barber et al 2021; Table S2). A) Counts of *Starships* with either 'insert' or 'flank' boundaries, which are derived directly from pairwise genome alignments against a putative insertion site. B) Counts of *Starships* with 'insert' or 'flank' boundaries plus those with 'extend' boundaries, which are derived from aligning genomic sequences to known *Starship* sequences. Insert and flank boundaries are associated with full-length *Starship* elements, while "extend" boundaries may be associated with either full-length *Starship* elements or element fragments.

Figure S2: Nucleotide alignments of representative *Starship* copies, +/- 100kb of flanking sequence, across all genomic regions where that *Starship* is found. Predicted functions of interest are annotated above the corresponding gene. Links between schematics represent alignable regions ≥1000bp and ≥90% nucleotide sequence identity. A) *Tardis*. B) *Gnosis*. C) *Janus*. D) *Osiris*. E) *Lamia*. F) *Logos*. G) *Nebuchadnezzar.*

Figure S3: A scatterplot depicting pairwise comparisons of single nucleotide polymorphism (SNP)-based Identity by State (IBS) and Jaccard similarity in high-confidence Starship presence/absence profiles between 151 Clade 1 strains from Lofgren et al. 2022 (Table S8).

Figure S4: Screenshots from the IGV genome browser showing deletions of *Nebuchadnezzar h1* among different isolates of the ATCC46645 strain sequenced by different research groups (regions depicted in red denote *Nebuchadnezzar h1*). Illumina short-reads of strain ATCC46645 sequenced by a different research group (accession: SRR7418935) mapped to the ATCC46645 genome sequenced with long read technology in this study. A zoom in of the genomic location of *Nebuchadnezzar h1* in the ATCC46645 long read assembly is shown (contig accession: ATCC46645-lr_scaffold15). Three short-read tracks indicate a deletion of the *Starship* (above). Note that more short reads are mapped than shown in the image. Track and color descriptions can be found in the IGV manual.

Figure S5: Donut charts summarizing the percentages of gene orthogroups in the core, accessory, singleton, and *Starship*-associated compartments of the *Aspergillus fumigatus* pangenome, derived from the 13 reference-quality *A. fumigatus* strains and the expanded set of 54 high and medium-confidence *Starships* (n = 1818 elements total; Table S11).

Figure S6: Iceberg plots summarizing the genomic locations of the single best BLASTp hits (≥90% identity, ≥33% query coverage) to the cargo genes from the type elements of the 20 high-confidence *Starships* in 519 *Aspergillus fumigatus* assemblies (Table S13). Each column represents a cargo gene. A) Results broken down by *Starship*, with columns arranged according to gene order within each *Starship*. B) Compiled results across all 20 *Starships*, with columns arranged according to the number of strains with BLASTp hits. Bars are colored according to the genomic location in which the BLASTp hits are found.

Figure S7: Barcharts summarizing the genotypes of segregating genomic regions associated with the 20 high-confidence elements in the *Aspergillus fumigatus* 519 strain population (Table S16). If an isolate did not have any Starships within a given region, it was assigned either an “empty” or “fragmented” genotype (Methods).

Figure S8: The putative target site of *Starship Tardis* (indicated with an arrow) occurs more often than you would expect by chance in the *Aspergillus fumigatus* genome. We estimated the copy numbers of all k-mers of length 10 in the Af293 reference genome and found that the k-mer of length 10 that corresponds to the putative target site of *Tardis* is present in high copy numbers that exceed the expected genome-wide frequency of k-mers of this length (>99.99th percentile).

Figure S9: *Starships* mobilize genes encoding diverse metabolic functions. Barcharts summarizing the presence of genes with metabolism-related COG (Clusters of Orthologous Groups) annotations in the 20 high-confidence *Starships* in the 519 *Aspergillus fumigatus* strain population (Table S18). The X-axis measures the percentage of copies of a given *Starship* that carry at least 1 gene with an annotation of interest.

Figure S10: The Biofilm Architecture Factor (*baf*) gene family is closely associated with diverse *Starships* in *Aspergillus fumigatus*. A maximum likelihood tree of *baf* sequences from the 519 *Aspergillus fumigatus* strain population. Branches with ≥80% SH-ALRT and ≥95% ultrafast bootstrap support are in bold. *Baf* sequences found in *Starships* have the corresponding *Starship* identification number appended to their right (Table S5). Sequences are color-coded according to 6 corresponding *baf* clades of interest, and a summary of all the *Starship* types found associated with each clade is printed on the right.

Figure S11: *Starships* are enriched in environmental and clinical strains. Barcharts summarizing the proportion of strains from 475/479 *Aspergillus fumigatus* genotyped strains with known isolation sources that have the 20 high-confidence *Starships*, broken up by isolation source. Fisher’s exact test P values for *Starship* enrichment across isolation source categories are shown above each bar (adjusted for multiple comparisons using the Benjamini Hochberg procedure; Table S20).

Figure S12: A heatmap of transcript abundances ($\log_{10}$ TPM) of *Starship* captain genes (A, C) and genes within the *hrmA*-associated cluster (HAC; B, D), collapsed across treatment replicates, for RNAseq studies from *A. fumigatus* CEA10 (A, B) and A1163 (C, D).

Figure S13: Heatmap of binary core transcript coverage across genes in *Starships* in strains Af293 (A), CEA10 (B) and A1163 (C). Genes that have a median core transcript coverage greater than 1 are shown here in blue, and values below 1 are shown in red.

Figure S14: Differentially expressed *Starship*-mobilized genes (DEGs) displayed in volcano plots across combinations of *A. fumigatus* strains and treatment categories. Differential expression based on singleton studies are shown as $\log_2$ fold-change ($\log_2$FC) in black with standard error

bars, whereas DEGs identified across multiple studies are represented with summarized $log_2FC$ values from a random effects model (REM) and coloured by values “sign-consistency”, the number of studies that reported differential gene expression in the positive (+1) or negative (-1) direction, centered around 0.

Figure S15: Distributions of TOM scores from the WGCNA constructed from Af293 *A. fumigatus* studies. TOM scores were separated into distinct categories for edges that connected any two genes within a *Starships*, between different *Starships*, between *Starships* and the rest of the genome, or between any two non-*Starship* genes in the genome were compared. These distributions were compared using a Wilcoxon test (p > 0.05 = “ns”; p < 0.05 = “*”, p < 0.01* = “**”, p < 0.001 = “***”, p < 0.0001 = “****”).

Figure S16: Heatmap displaying the correlation values for eigengenes in each module and their association with samples from a specific study (labeled with BioProject IDs), or different levels of experimental treatment category across studies. Cells of the heatmap are coloured by the correlation value, as well as labeled in each cell. P-values from a pairwise comparison between observations (*pdist*) are shown in parentheses.

Figure S1

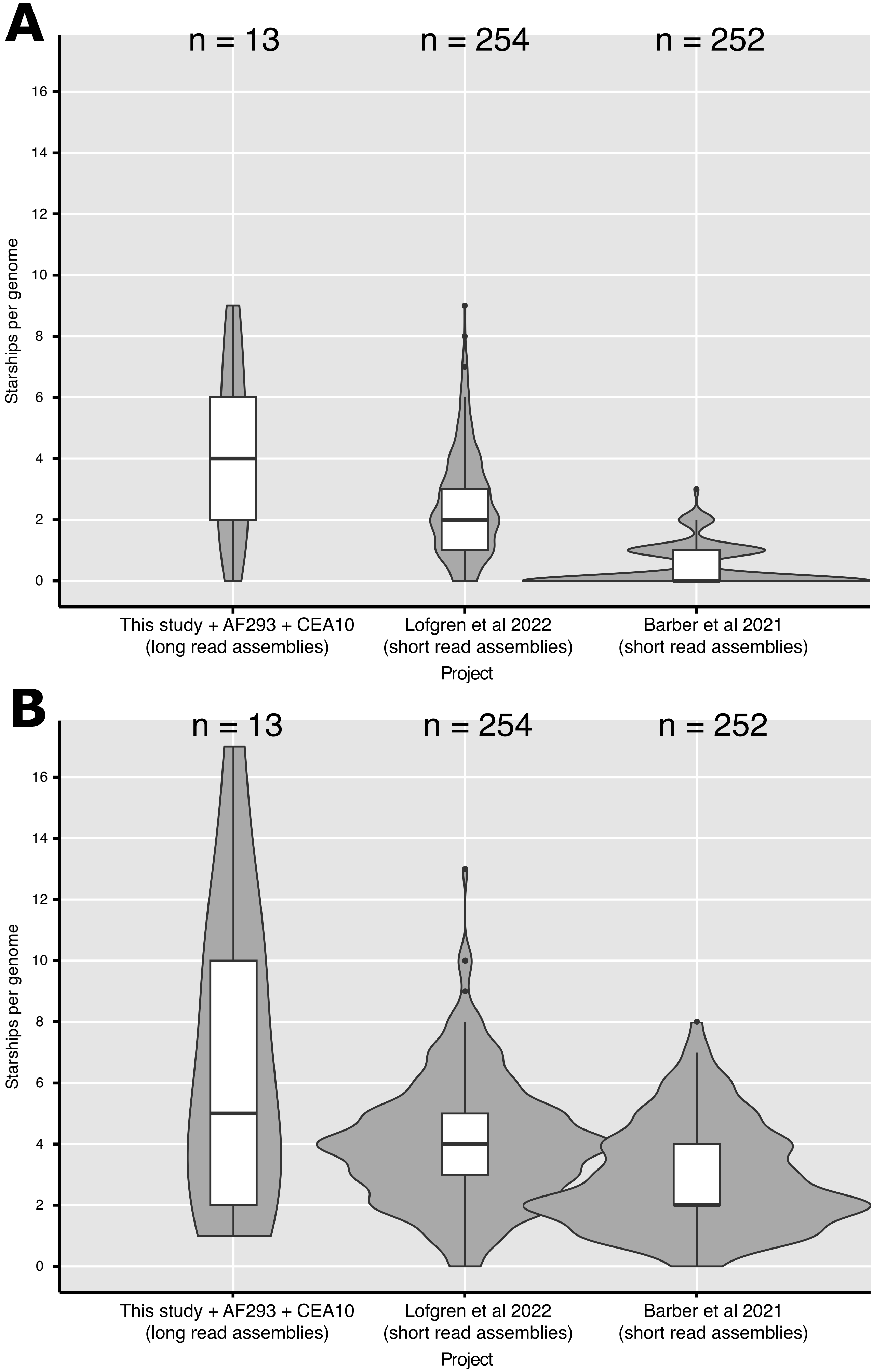
A
n = 13
n = 254
n = 252
Starships per genome
0
2
4
6
8
10
12
14
16
This study + AF293 + CEA10
(long read assemblies)
Lofgren et al 2022
(short read assemblies)
Barber et al 2021
(short read assemblies)
Project
B
n = 13
n = 254
n = 252
Starships per genome
0
2
4
6
8
10
12
14
16
This study + AF293 + CEA10
(long read assemblies)
Lofgren et al 2022
(short read assemblies)
Barber et al 2021
(short read assemblies)
Project

# Figure S2A

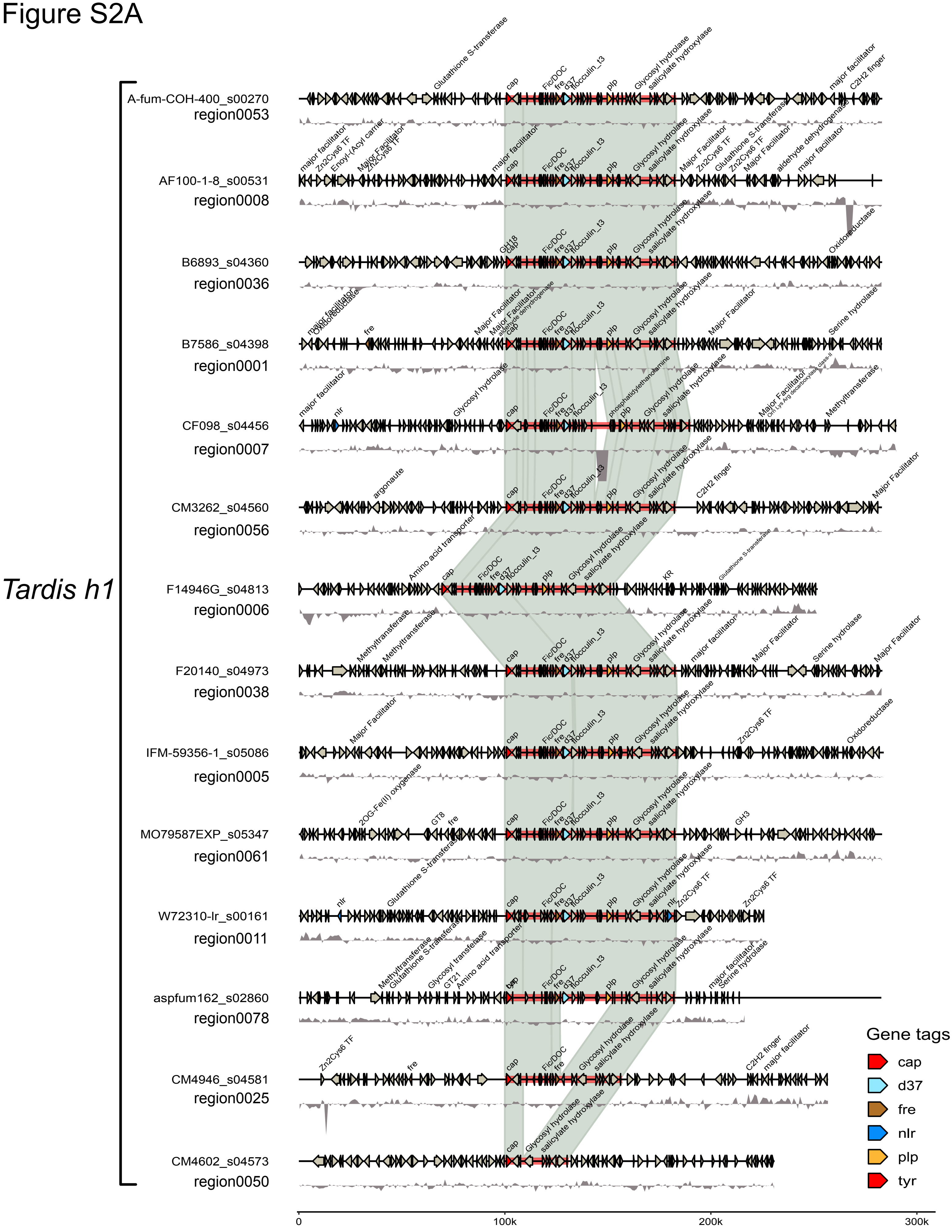
Tardis h1
A-fum-COH-400_s00270
region0053
AF100-1-8_s00531
region0008
B6893_s04360
region0036
B7586_s04398
region0001
CF098_s04456
region0007
CM3262_s04560
region0056
F14946G_s04813
region0006
F20140_s04973
region0038
IFM-59356-1_s05086
region0005
MO79587EXP_s05347
region0061
W72310-Ir_s00161
region0011
aspfum162_s02860
region0078
CM4946_s04581
region0025
CM4602_s04573
region0050
0
100k
200k
300k
Gene tags
cap
d37
fre
nlr
plp
tyr

# Figure S2B

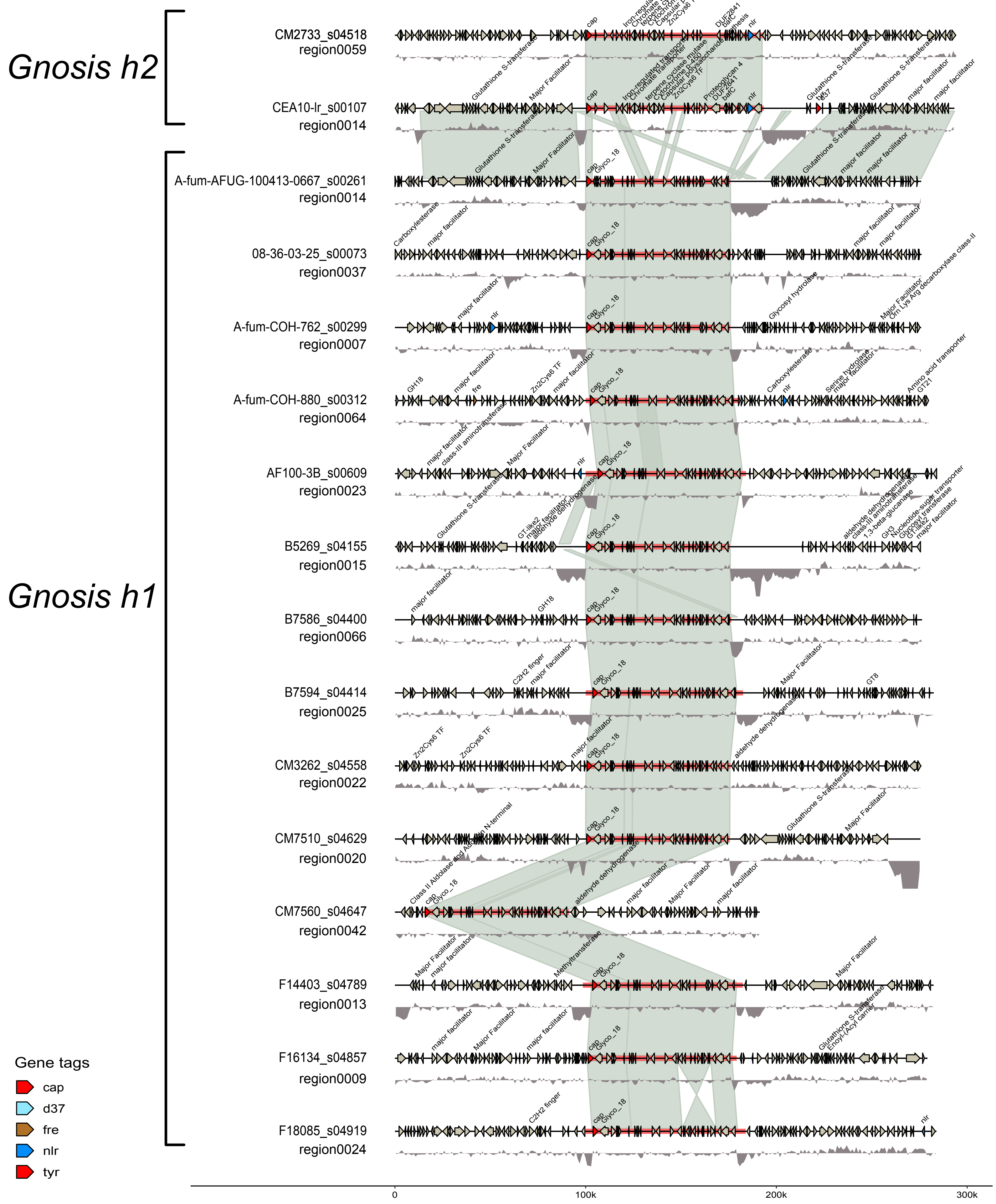

Gnosis h2
Gnosis h1
CM2733_s04518
region0059
CEA10-lr_s00107
region0014
A-fum-AFUG-100413-0667_s00261
region0014
08-36-03-25_s00073
region0037
A-fum-COH-762_s00299
region0007
A-fum-COH-880_s00312
region0064
AF100-3B_s00609
region0023
B5269_s04155
region0015
B7586_s04400
region0066
B7594_s04414
region0025
CM3262_s04558
region0022
CM7510_s04629
region0020
CM7560_s04647
region0042
F14403_s04789
region0013
F16134_s04857
region0009
F18085_s04919
region0024
Gene tags
cap
d37
fre
nlr
tyr
0
100k
200k
300k

# Figure S2C

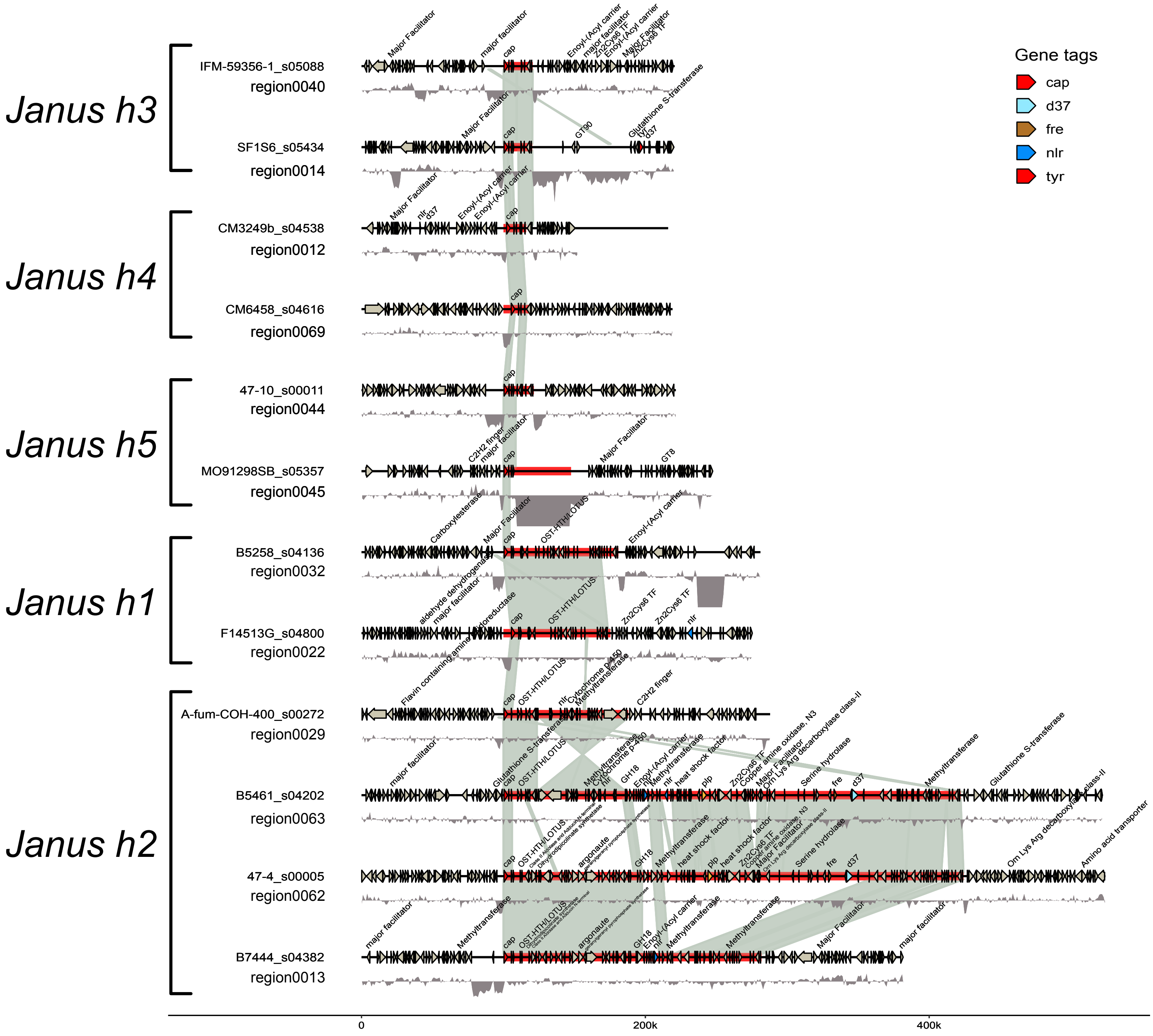

Janus h3
IFM-59356-1_s05088
region0040
SF1S6_s05434
region0014
Janus h4
CM3249b_s04538
region0012
CM6458_s04616
region0069
Janus h5
47-10_s00011
region0044
MO91298SB_s05357
region0045
Janus h1
B5258_s04136
region0032
F14513G_s04800
region0022
Janus h2
A-fum-COH-400_s00272
region0029
B5461_s04202
region0063
47-4_s00005
region0062
B7444_s04382
region0013
Gene tags
cap
d37
fre
nlr
tyr
0
200k
400k

# Figure S2D

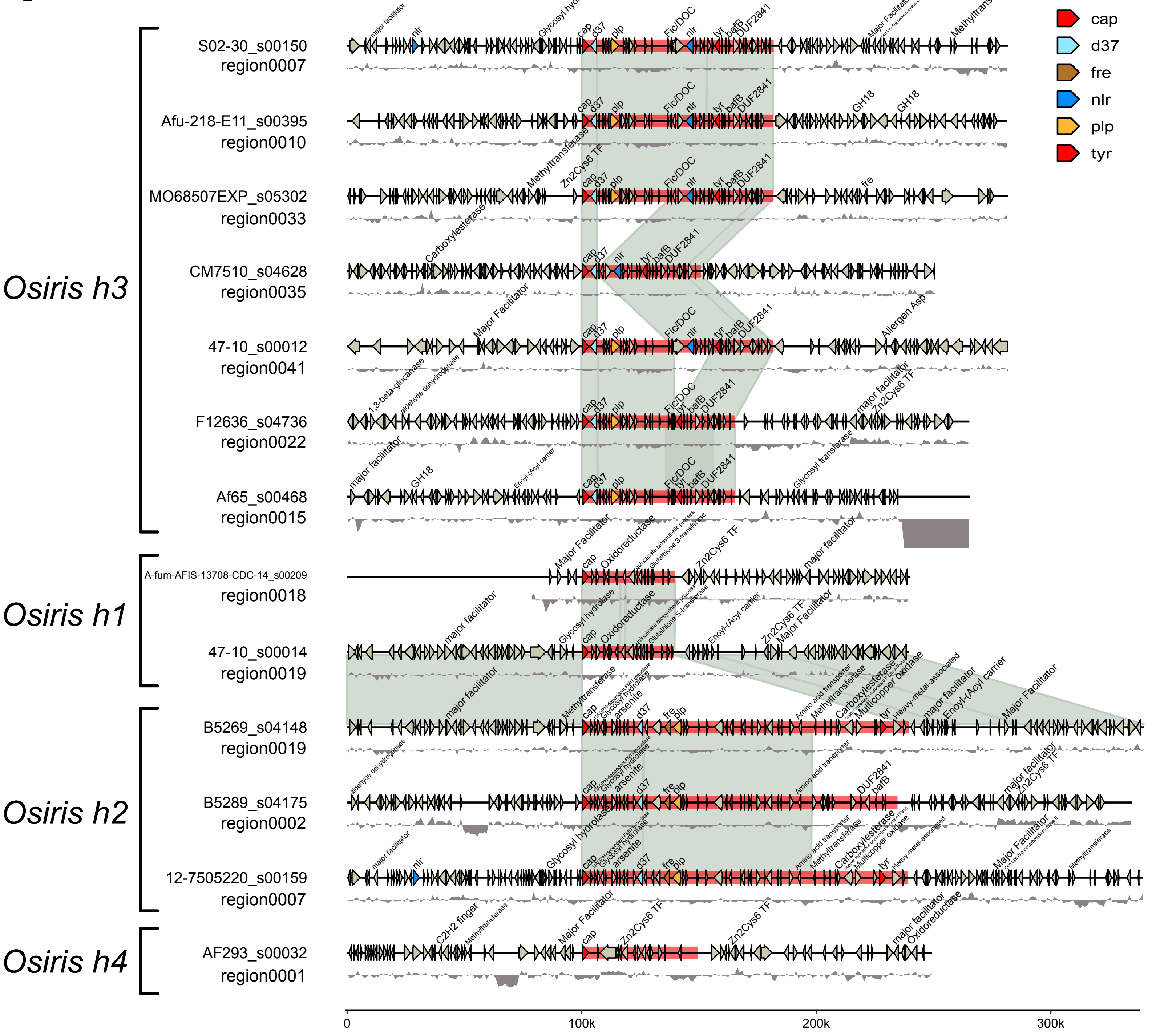
Gene tags
cap
d37
fre
nlr
plp
tyr
Osiris h3
S02-30_s00150
region0007
Afu-218-E11_s00395
region0010
MO68507EXP_s05302
region0033
CM7510_s04628
region0035
47-10_s00012
region0041
F12636_s04736
region0022
Af65_s00468
region0015
Osiris h1
A-fum-AFIS-13708-CDC-14_s00209
region0018
47-10_s00014
region0019
Osiris h2
B5269_s04148
region0019
B5289_s04175
region0002
12-7505220_s00159
region0007
Osiris h4
AF293_s00032
region0001
0
100k
200k
300k

# Figure S2E

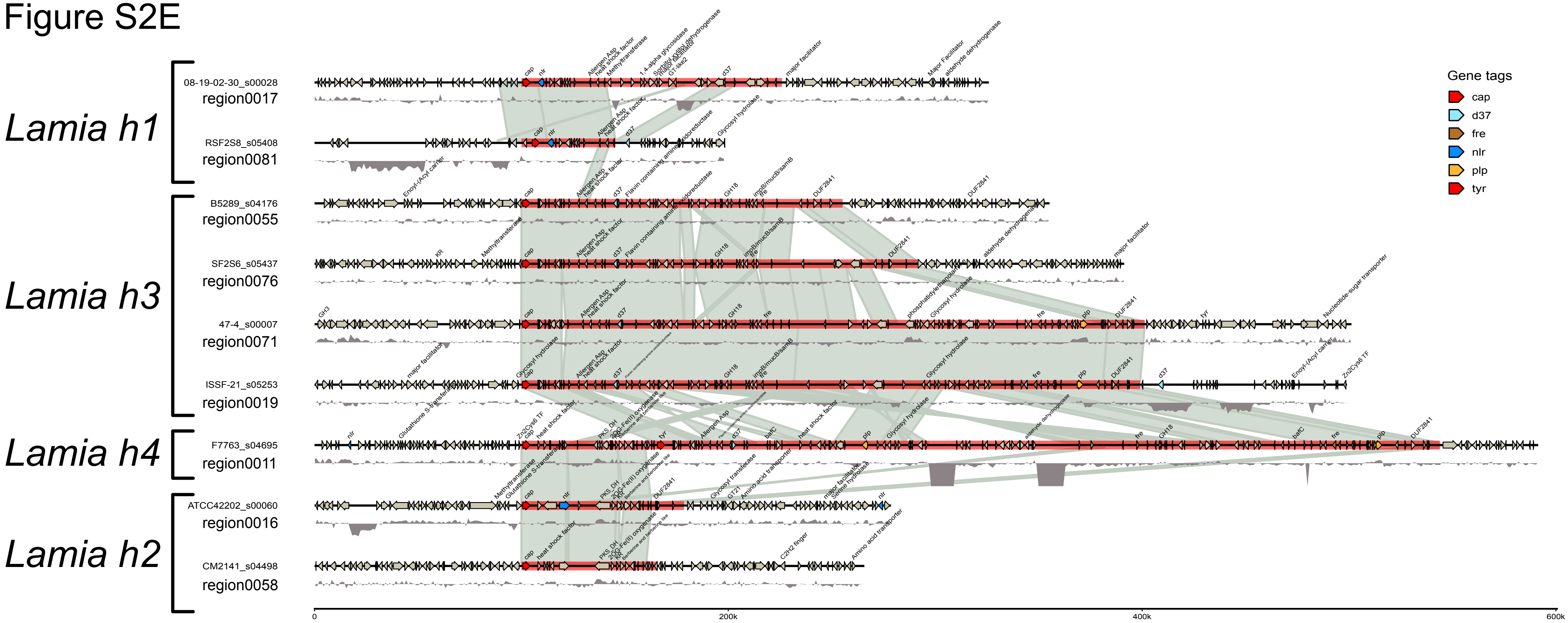

Lamia h1
08-19-02-30_s00028
region0017
RSF2S8_s05408
region0081
Lamia h3
B5289_s04176
region0055
SF2S6_s05437
region0076
47-4_s00007
region0071
ISSF-21_s05253
region0019
Lamia h4
F7763_s04695
region0011
Lamia h2
ATCC42202_s00060
region0016
CM2141_s04498
region0058
0
200k
400k
600k
Gene tags
cap
d37
fre
nlr
plp
tyr

# Figure S2F

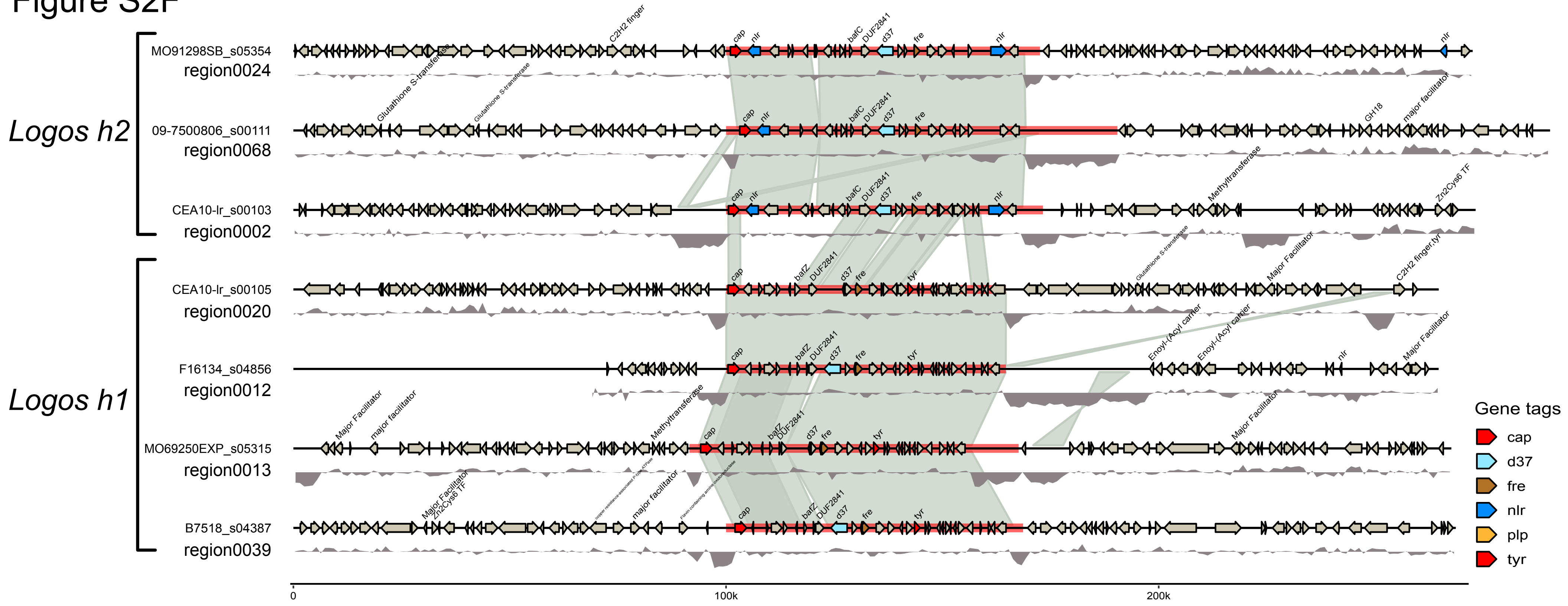

Logos h2
Logos h1
MO91298SB_s05354
region0024
09-7500806_s00111
region0068
CEA10-lr_s00103
region0002
CEA10-lr_s00105
region0020
F16134_s04856
region0012
MO69250EXP_s05315
region0013
B7518_s04387
region0039
cap
nlr
bafC
bafZ
DUF2841
d37
fre
tyr
C2H2 finger
Glutathione S-transferase
GH18
major facilitator
Major Facilitator
Methyltransferase
Zn2Cys6 TF
Enoyl-(Acyl carrier
C2H2 finger,tyr
0
100k
200k
Gene tags
cap
d37
fre
nlr
plp
tyr

Figure S2G

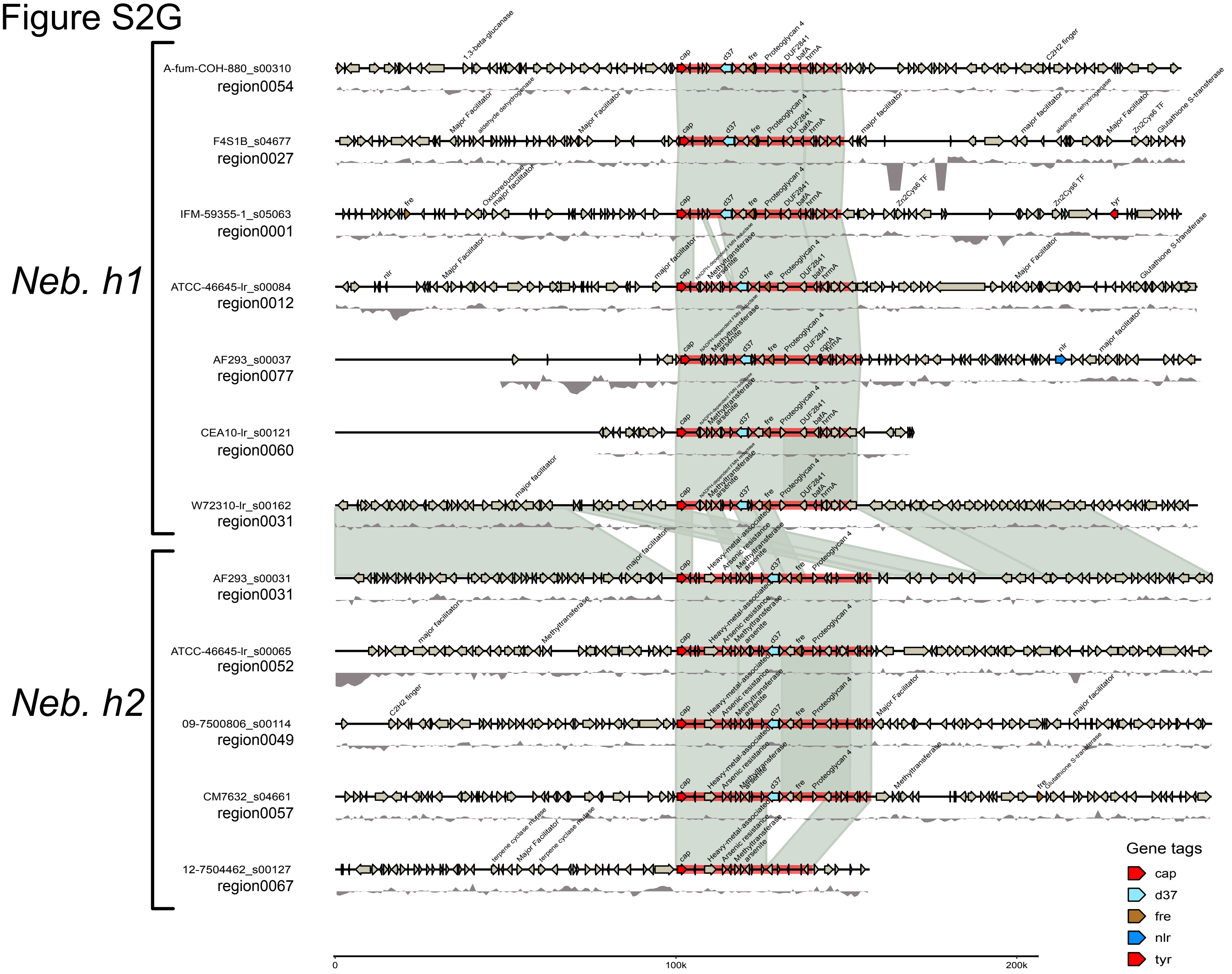

Neb. h1
A-fum-COH-880_s00310
region0054
F4S1B_s04677
region0027
IFM-59355-1_s05063
region0001
ATCC-46645-lr_s00084
region0012
AF293_s00037
region0077
CEA10-lr_s00121
region0060
W72310-lr_s00162
region0031
Neb. h2
AF293_s00031
region0031
ATCC-46645-lr_s00065
region0052
09-7500806_s00114
region0049
CM7632_s04661
region0057
12-7504462_s00127
region0067
0
100k
200k
Gene tags
cap
d37
fre
nlr
tyr

Figure S3

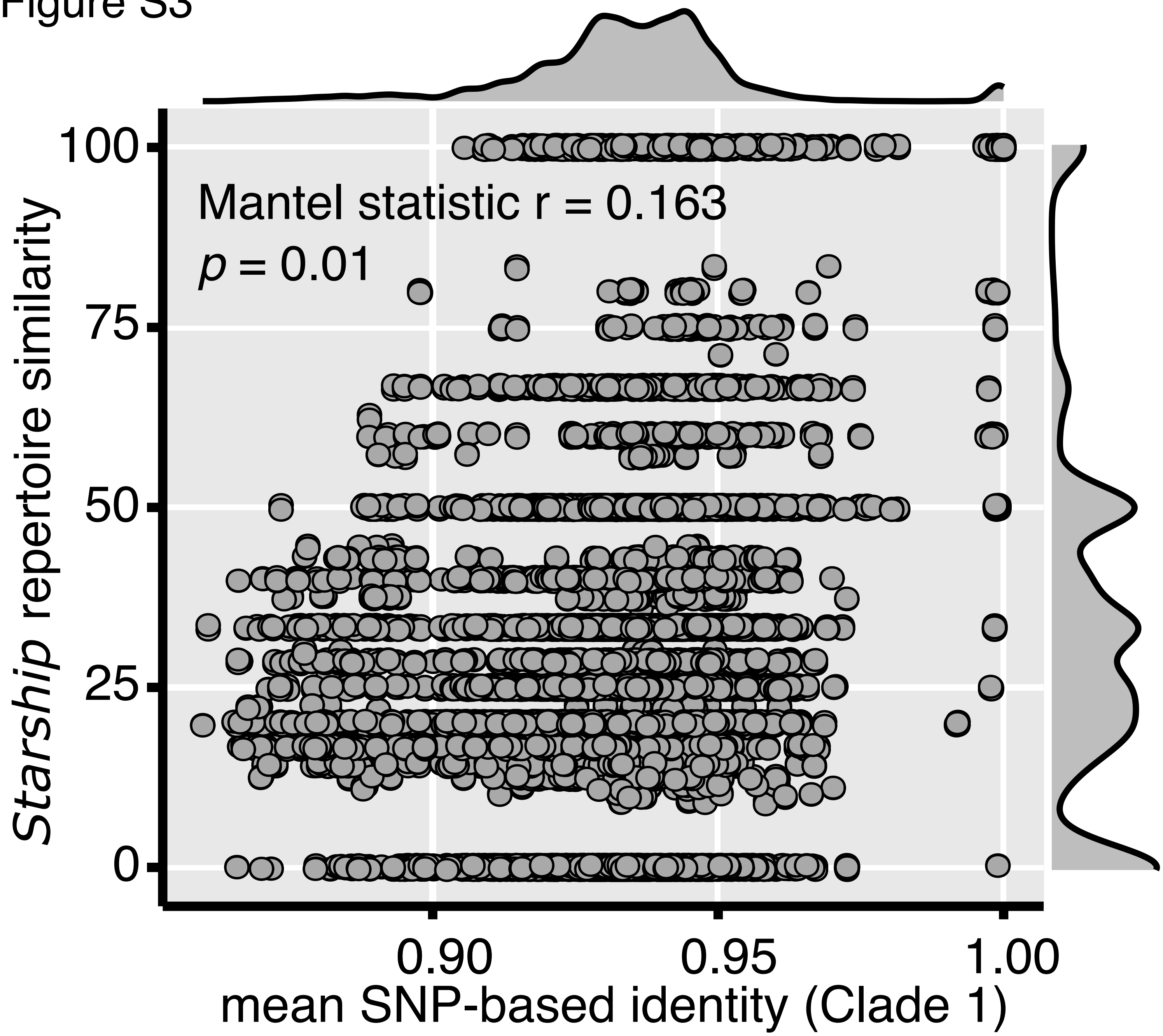
Mantel statistic r = 0.163
*p* = 0.01
*Starship* repertoire similarity
100
75
50
25
0
0.90
0.95
1.00
mean SNP-based identity (Clade 1)

# Figure S4

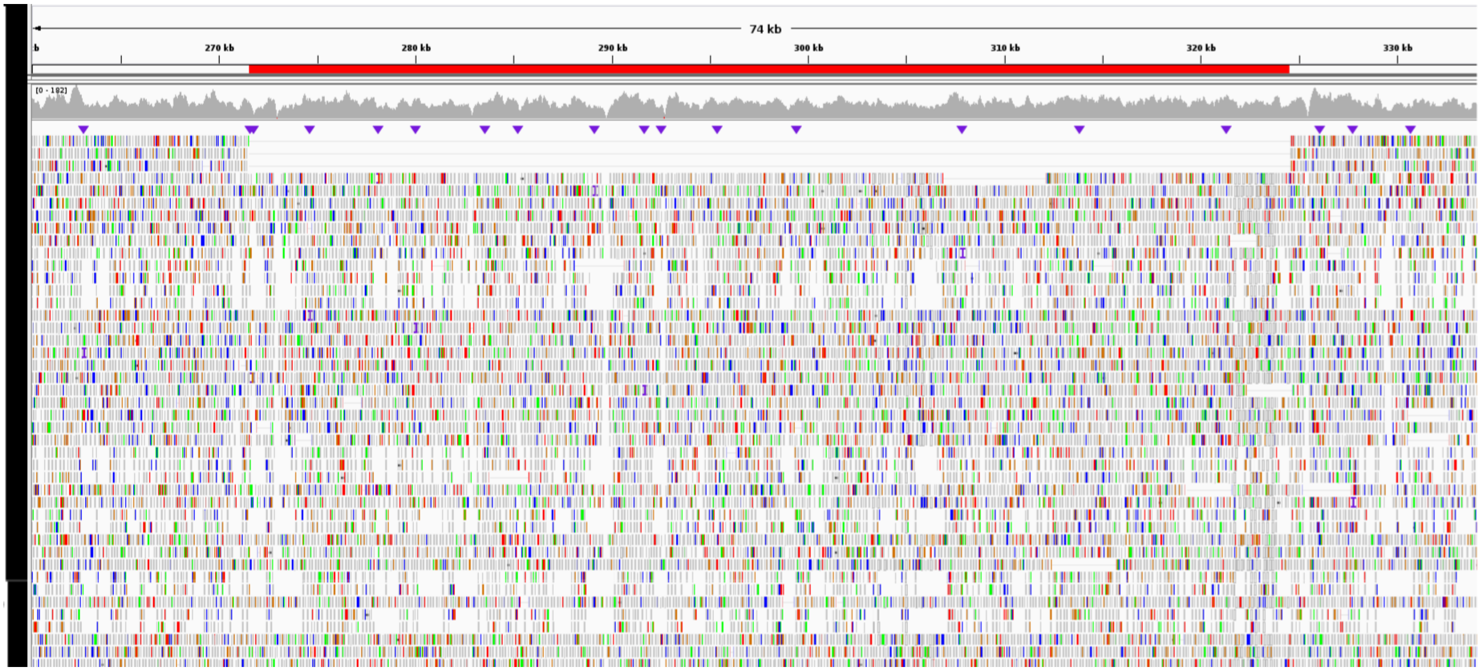

Figure S5

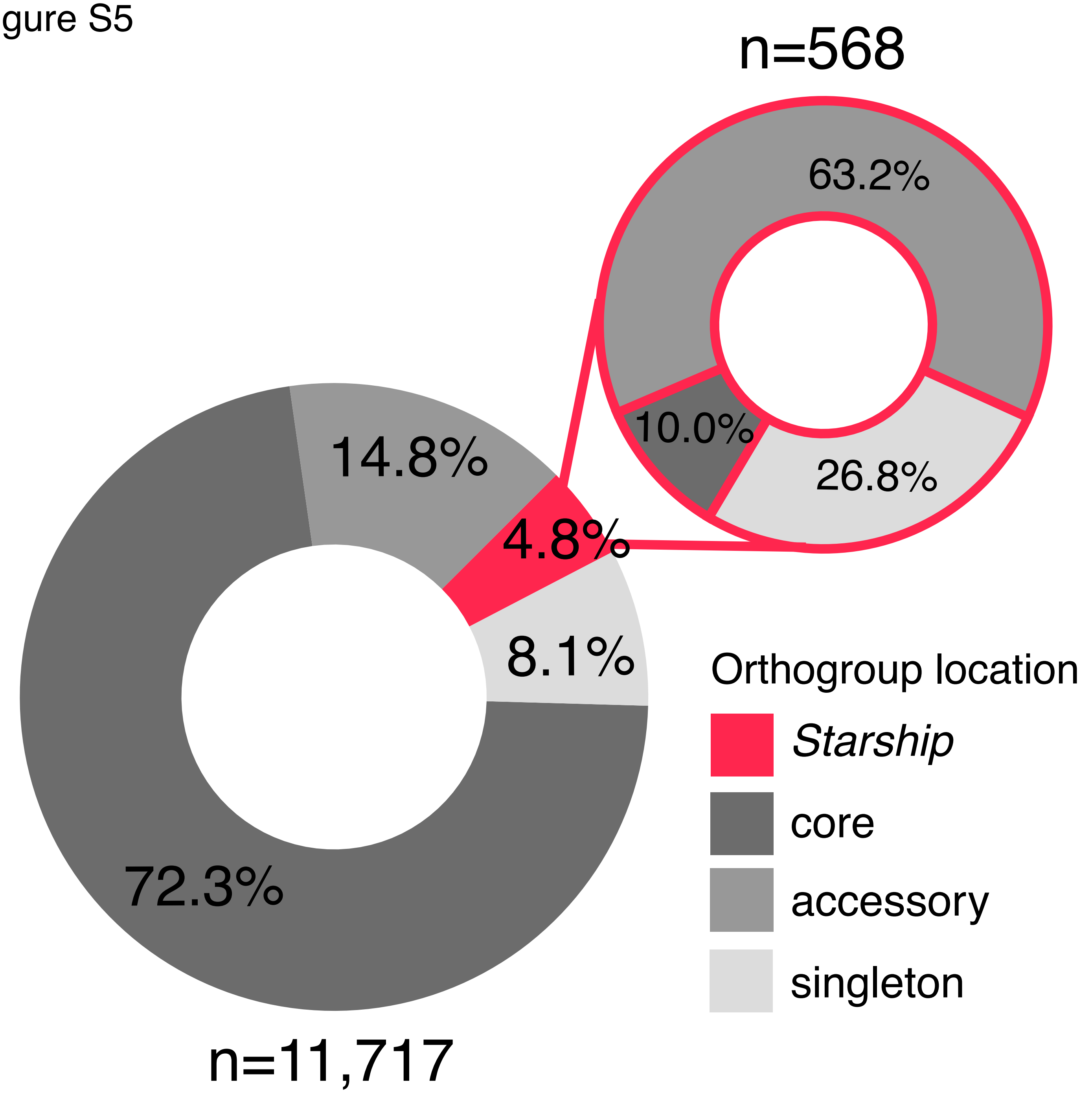
n=568
63.2%
10.0%
26.8%
14.8%
4.8%
8.1%
72.3%
n=11,717
Orthogroup location
Starship
core
accessory
singleton

# Figure S6

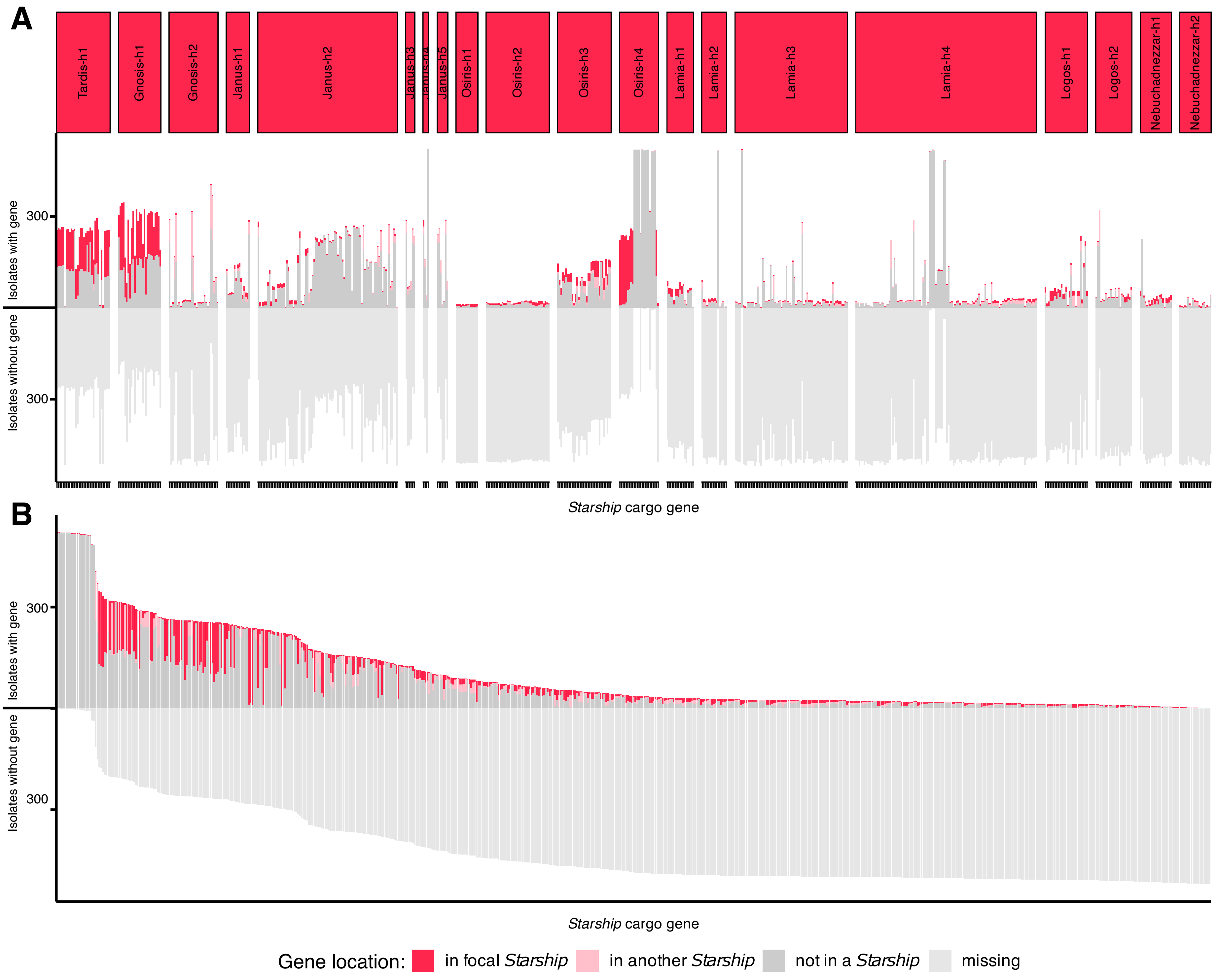
A
Tardis-h1
Gnosis-h1
Gnosis-h2
Janus-h1
Janus-h2
Janus-h3
Janus-h4
Janus-h5
Osiris-h1
Osiris-h2
Osiris-h3
Osiris-h4
Lamia-h1
Lamia-h2
Lamia-h3
Lamia-h4
Logos-h1
Logos-h2
Nebuchadnezzar-h1
Nebuchadnezzar-h2
Isolates with gene
Isolates without gene
300
300
Starship cargo gene
B
Isolates with gene
Isolates without gene
300
300
Starship cargo gene
Gene location:
in focal Starship
in another Starship
not in a Starship
missing

# Figure S7

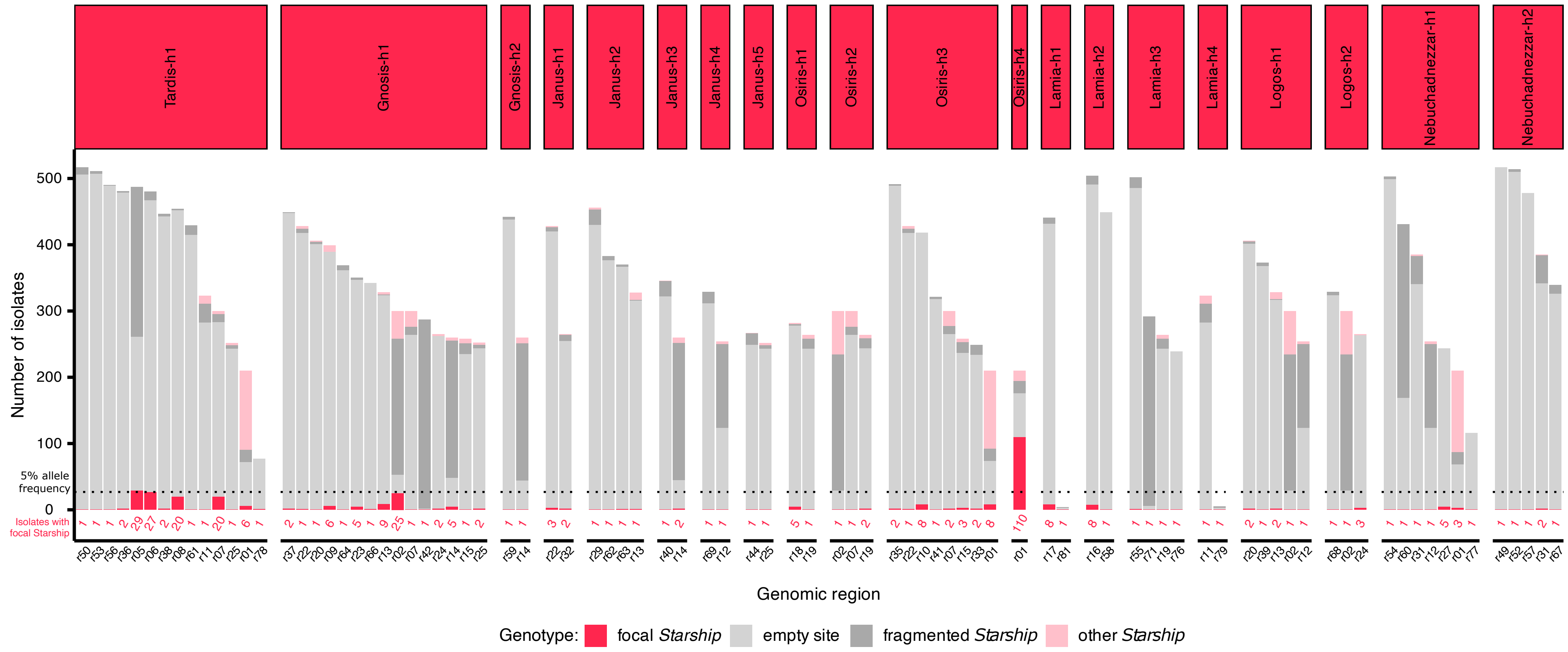

Tardis-h1
Gnosis-h1
Gnosis-h2
Janus-h1
Janus-h2
Janus-h3
Janus-h4
Janus-h5
Osiris-h1
Osiris-h2
Osiris-h3
Osiris-h4
Lamia-h1
Lamia-h2
Lamia-h3
Lamia-h4
Logos-h1
Logos-h2
Nebuchadnezzar-h1
Nebuchadnezzar-h2
Number of isolates
5% allele frequency
Isolates with focal Starship
Genomic region
Genotype: focal *Starship* empty site fragmented *Starship* other *Starship*

Figure S8

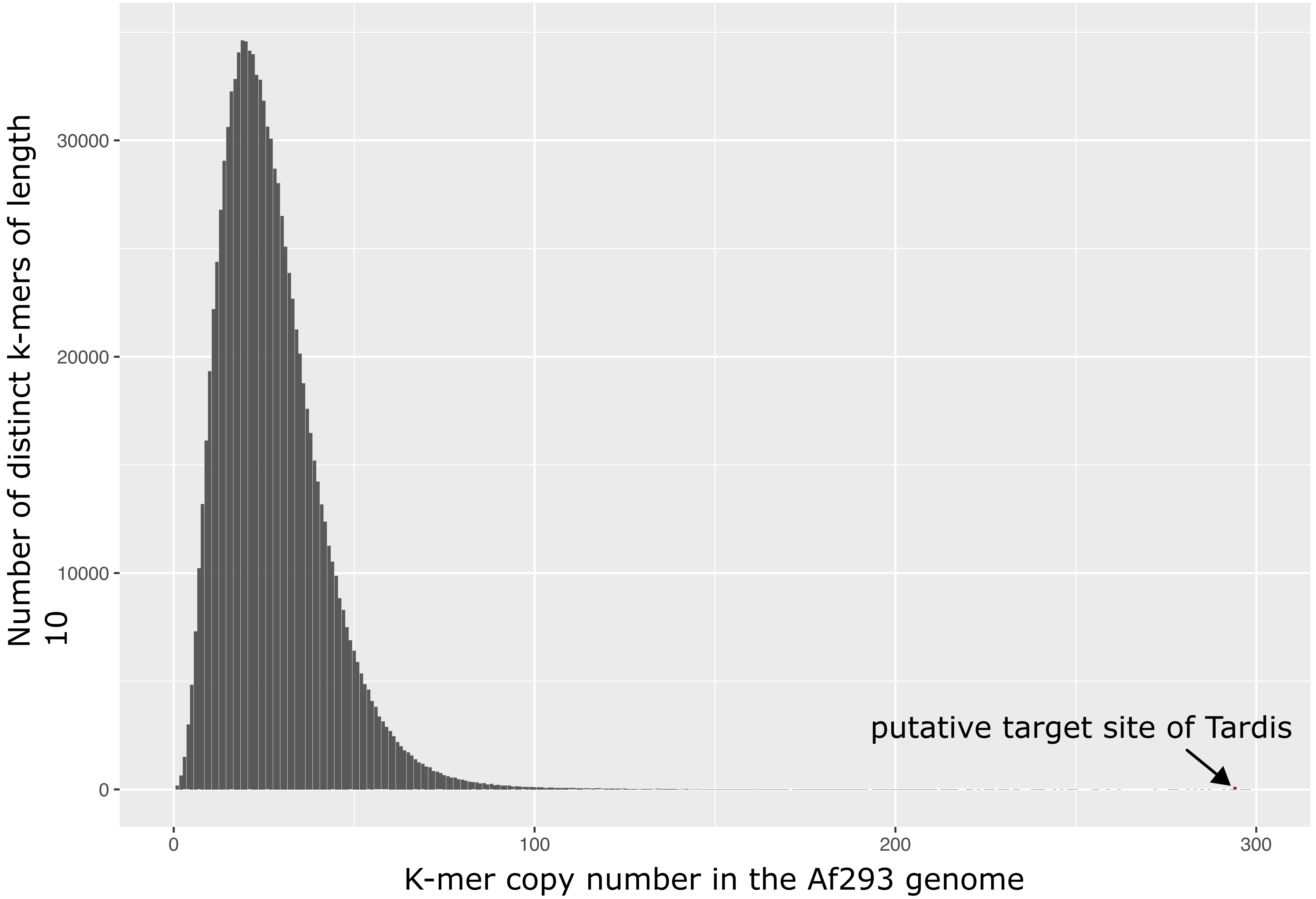

Number of distinct k-mers of length 10
30000
20000
10000
0
0
100
200
300
K-mer copy number in the Af293 genome
putative target site of Tardis

# Figure S9

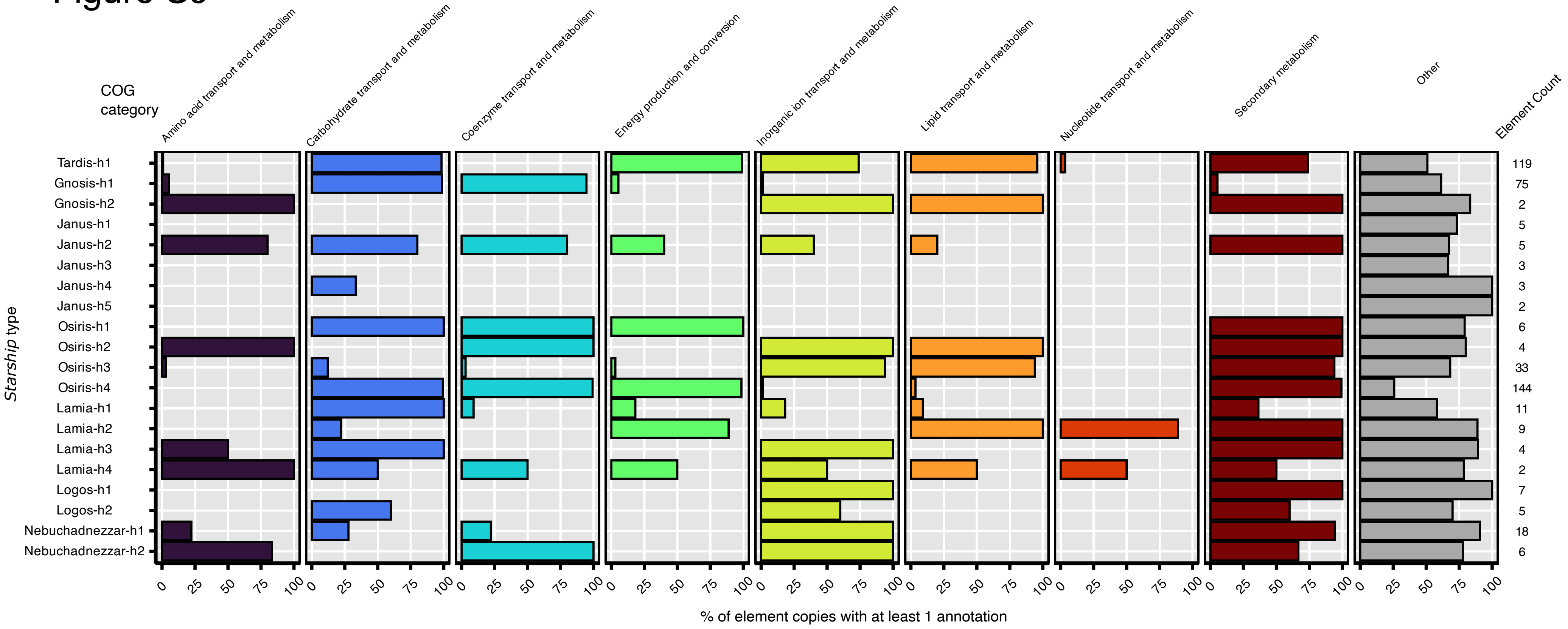

COG category
Amino acid transport and metabolism
Carbohydrate transport and metabolism
Coenzyme transport and metabolism
Energy production and conversion
Inorganic ion transport and metabolism
Lipid transport and metabolism
Nucleotide transport and metabolism
Secondary metabolism
Other
Element Count
Starship type
Tardis-h1 119
Gnosis-h1 75
Gnosis-h2 2
Janus-h1 5
Janus-h2 5
Janus-h3 3
Janus-h4 3
Janus-h5 2
Osiris-h1 6
Osiris-h2 4
Osiris-h3 33
Osiris-h4 144
Lamia-h1 11
Lamia-h2 9
Lamia-h3 4
Lamia-h4 2
Logos-h1 7
Logos-h2 5
Nebuchadnezzar-h1 18
Nebuchadnezzar-h2 6
0 25 50 75 100
% of element copies with at least 1 annotation

# Figure S10

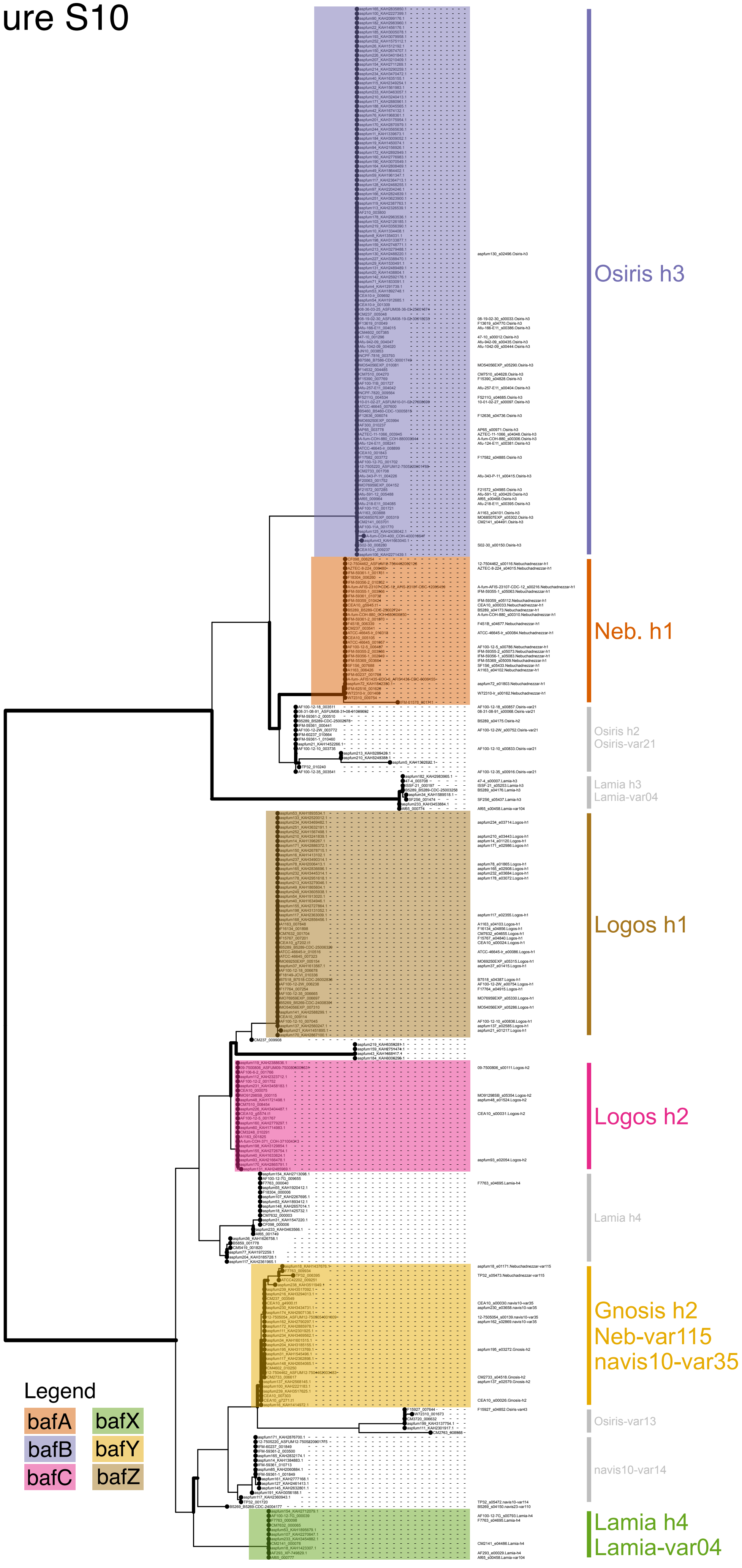
Osiris h3
Neb. h1
Osiris h2
Osiris-var21
Lamia h3
Lamia-var04
Logos h1
Logos h2
Lamia h4
Gnosis h2
Neb-var115
navis10-var35
Osiris-var13
navis10-var14
Lamia h4
Lamia-var04
Legend
bafA
bafX
bafB
bafY
bafC
bafZ

Figure S11

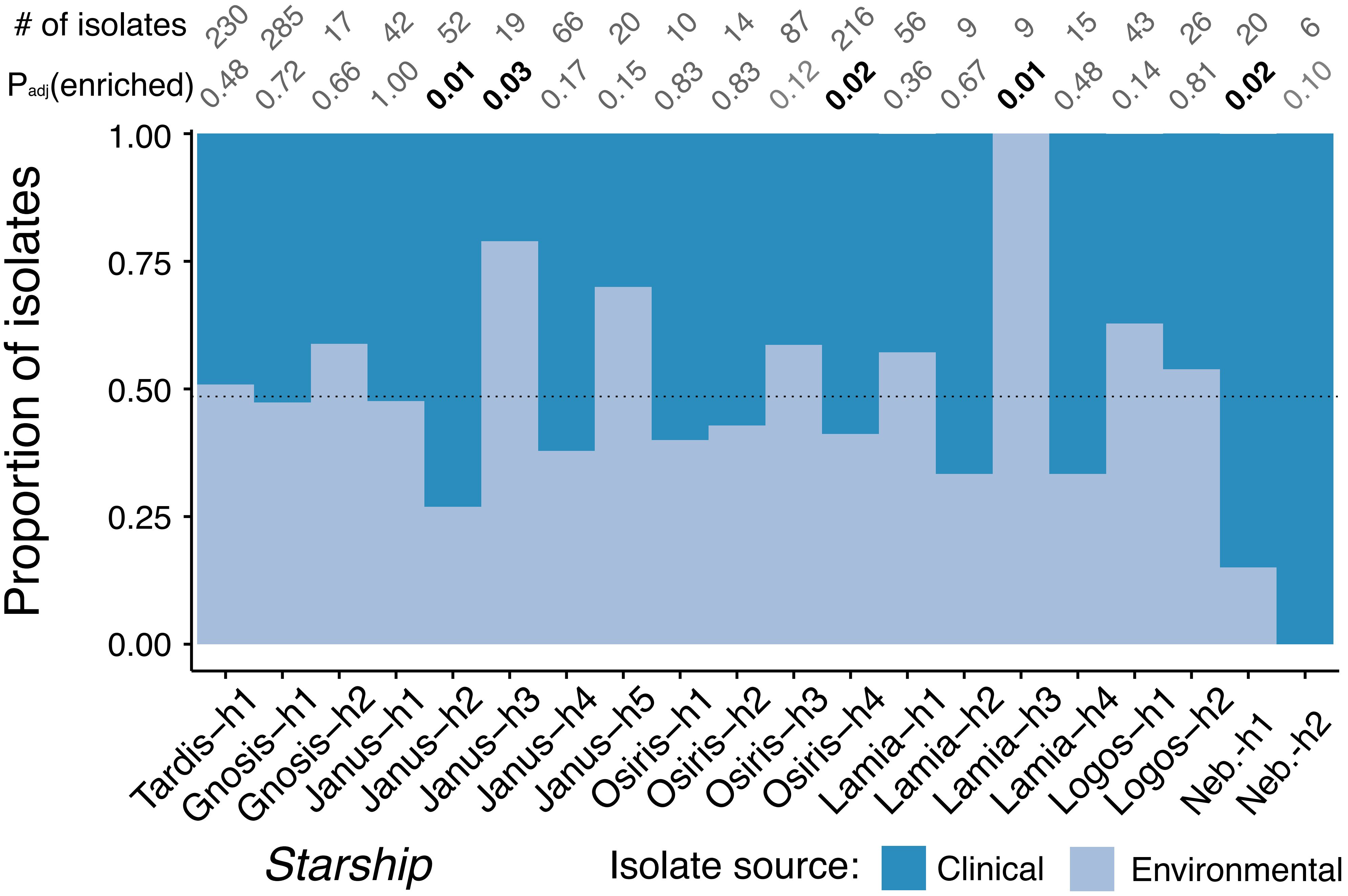

# of isolates
230 285 17 42 52 19 66 20 10 14 87 216 56 9 9 15 43 26 20 6
Padj(enriched)
0.48 0.72 0.66 1.00 0.01 0.03 0.17 0.15 0.83 0.83 0.12 0.02 0.36 0.67 0.01 0.48 0.14 0.81 0.02 0.10
Proportion of isolates
1.00
0.75
0.50
0.25
0.00
Tardis–h1
Gnosis–h1
Gnosis–h2
Janus–h1
Janus–h2
Janus–h3
Janus–h4
Janus–h5
Osiris–h1
Osiris–h2
Osiris–h3
Osiris–h4
Lamia–h1
Lamia–h2
Lamia–h3
Lamia–h4
Logos–h1
Logos–h2
Neb.-h1
Neb.-h2
Starship
Isolate source:
Clinical
Environmental

Figure S12

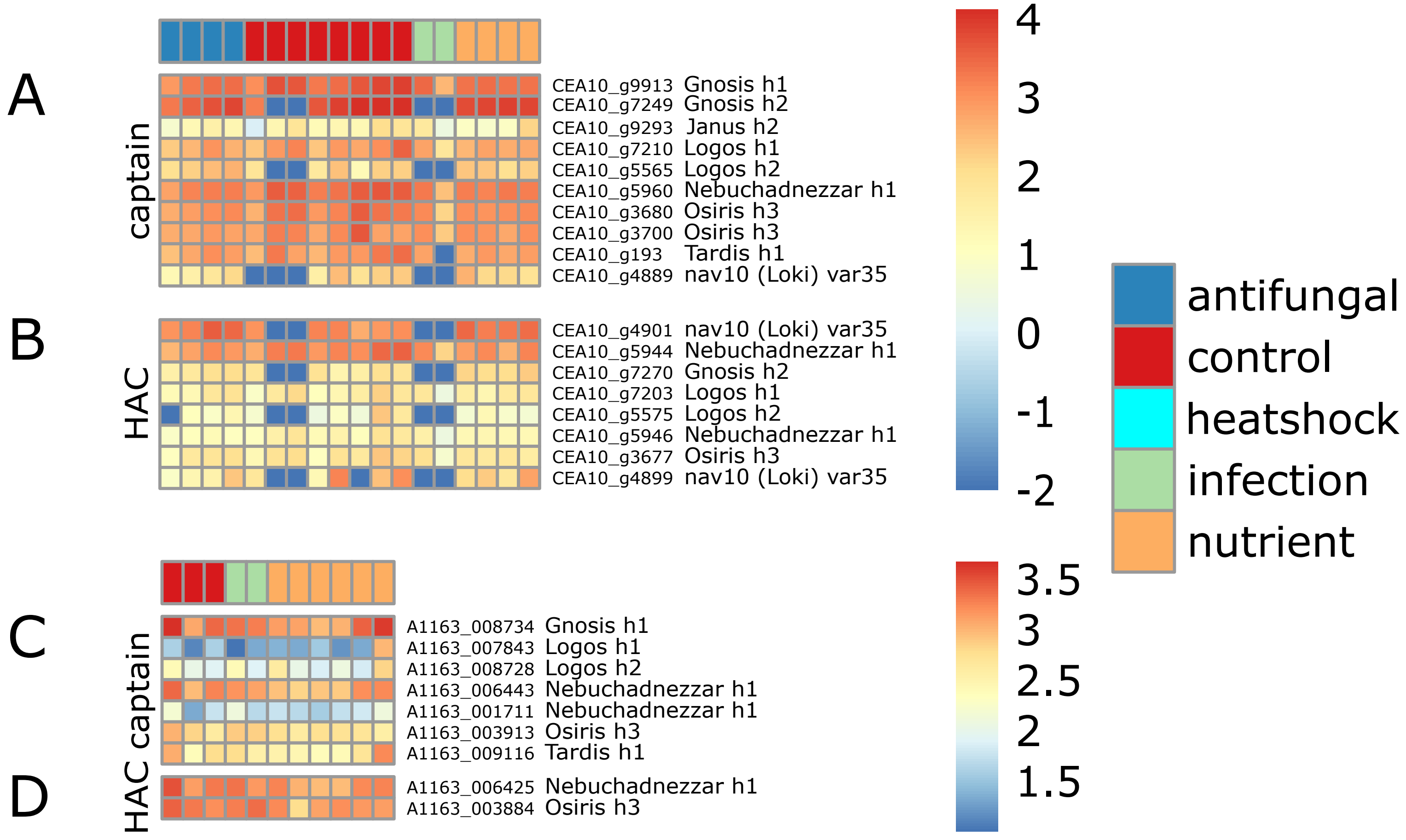
A
captain
CEA10_g9913 Gnosis h1
CEA10_g7249 Gnosis h2
CEA10_g9293 Janus h2
CEA10_g7210 Logos h1
CEA10_g5565 Logos h2
CEA10_g5960 Nebuchadnezzar h1
CEA10_g3680 Osiris h3
CEA10_g3700 Osiris h3
CEA10_g193 Tardis h1
CEA10_g4889 nav10 (Loki) var35
B
HAC
CEA10_g4901 nav10 (Loki) var35
CEA10_g5944 Nebuchadnezzar h1
CEA10_g7270 Gnosis h2
CEA10_g7203 Logos h1
CEA10_g5575 Logos h2
CEA10_g5946 Nebuchadnezzar h1
CEA10_g3677 Osiris h3
CEA10_g4899 nav10 (Loki) var35
4
3
2
1
0
-1
-2
antifungal
control
heatshock
infection
nutrient
C
HAC captain
A1163_008734 Gnosis h1
A1163_007843 Logos h1
A1163_008728 Logos h2
A1163_006443 Nebuchadnezzar h1
A1163_001711 Nebuchadnezzar h1
A1163_003913 Osiris h3
A1163_009116 Tardis h1
D
A1163_006425 Nebuchadnezzar h1
A1163_003884 Osiris h3
3.5
3
2.5
2
1.5

# Figure S13

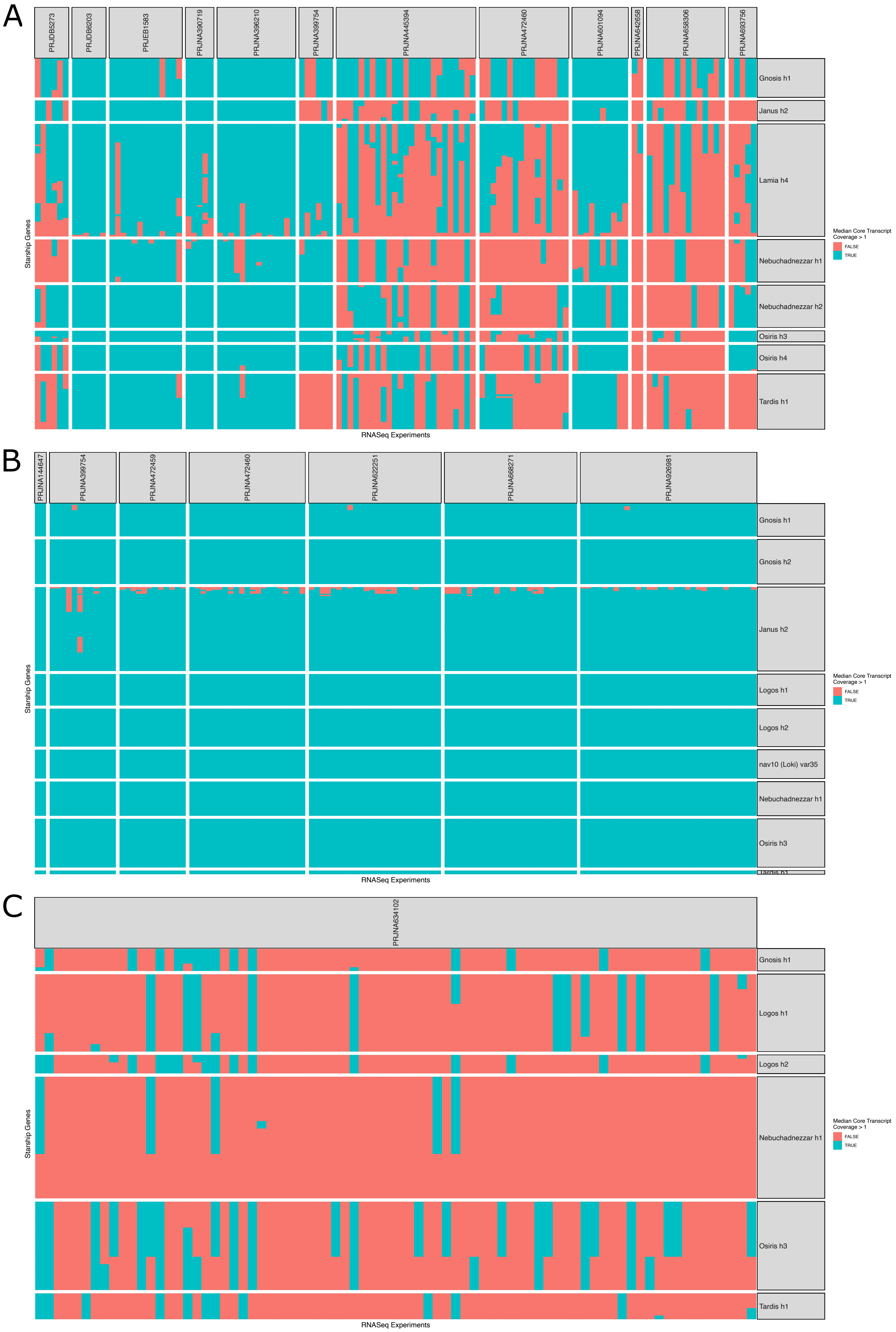
A
PRJDB5273
PRJDB6203
PRJEB1583
PRJNA390719
PRJNA396210
PRJNA399754
PRJNA445394
PRJNA472460
PRJNA601094
PRJNA642658
PRJNA658306
PRJNA693756
Gnosis h1
Janus h2
Lamia h4
Nebuchadnezzar h1
Nebuchadnezzar h2
Osiris h3
Osiris h4
Tardis h1
Starship Genes
RNASeq Experiments
Median Core Transcript Coverage > 1
FALSE
TRUE
B
PRJNA144647
PRJNA399754
PRJNA472459
PRJNA472460
PRJNA622251
PRJNA668271
PRJNA926981
Gnosis h1
Gnosis h2
Janus h2
Logos h1
Logos h2
nav10 (Loki) var35
Nebuchadnezzar h1
Osiris h3
Starship Genes
RNASeq Experiments
Median Core Transcript Coverage > 1
FALSE
TRUE
C
PRJNA634102
Gnosis h1
Logos h1
Logos h2
Nebuchadnezzar h1
Osiris h3
Tardis h1
Starship Genes
RNASeq Experiments
Median Core Transcript Coverage > 1
FALSE
TRUE

# Figure S14

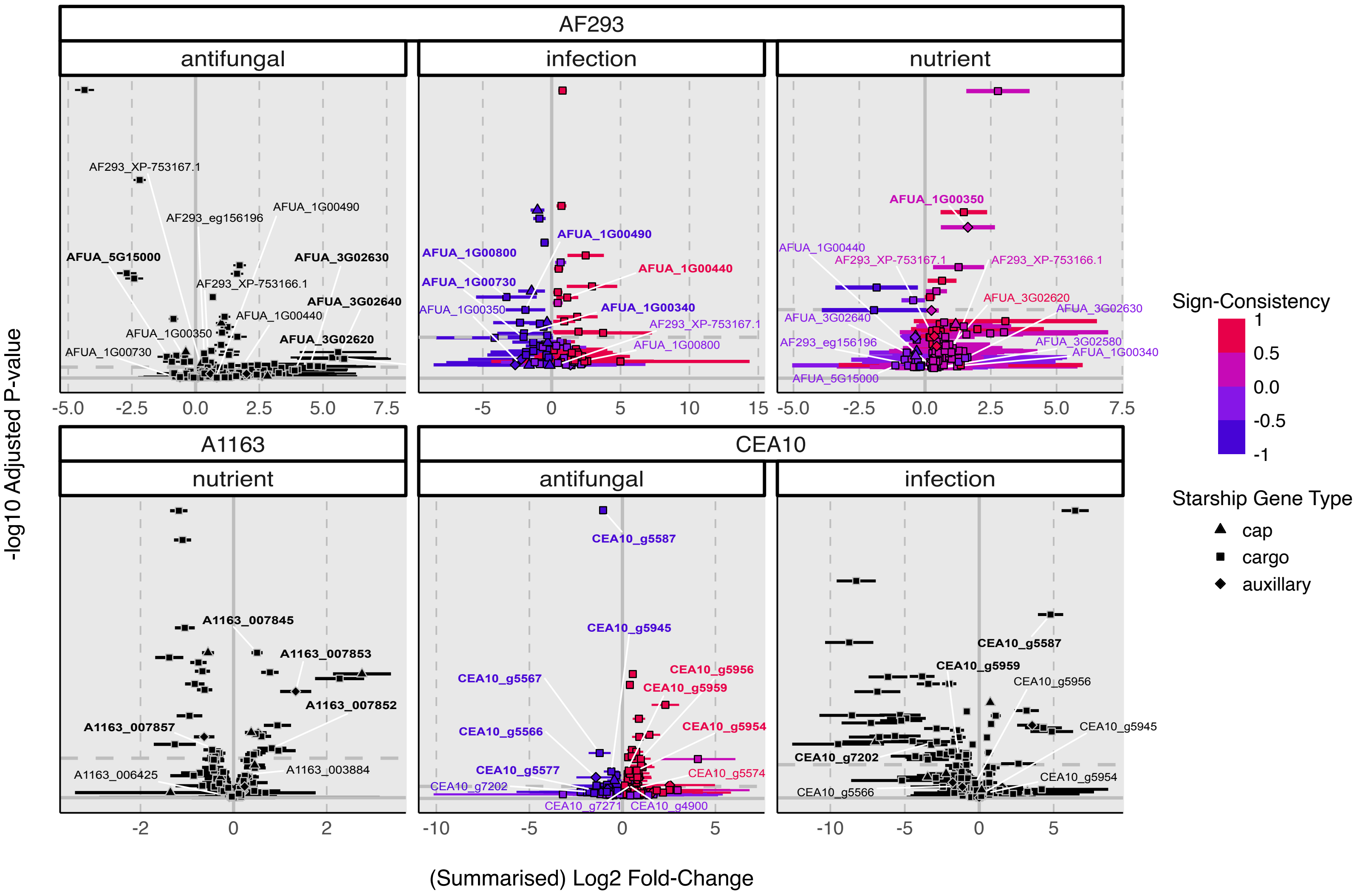

AF293
antifungal
infection
nutrient
A1163
nutrient
CEA10
antifungal
infection
-log10 Adjusted P-value
(Summarised) Log2 Fold-Change
Sign-Consistency
1
0.5
0.0
-0.5
-1
Starship Gene Type
cap
cargo
auxillary
AF293_XP-753167.1
AF293_eg156196
AFUA_1G00490
AFUA_5G15000
AFUA_3G02630
AF293_XP-753166.1
AFUA_3G02640
AFUA_1G00440
AFUA_1G00350
AFUA_3G02620
AFUA_1G00730
AFUA_1G00800
AFUA_1G00340
AFUA_3G02580
AFUA_5G15000
A1163_007845
A1163_007853
A1163_007852
A1163_007857
A1163_006425
A1163_003884
CEA10_g5587
CEA10_g5945
CEA10_g5956
CEA10_g5959
CEA10_g5567
CEA10_g5566
CEA10_g5954
CEA10_g5577
CEA10_g5574
CEA10_g7202
CEA10_g7271
CEA10_g4900

# Figure S15

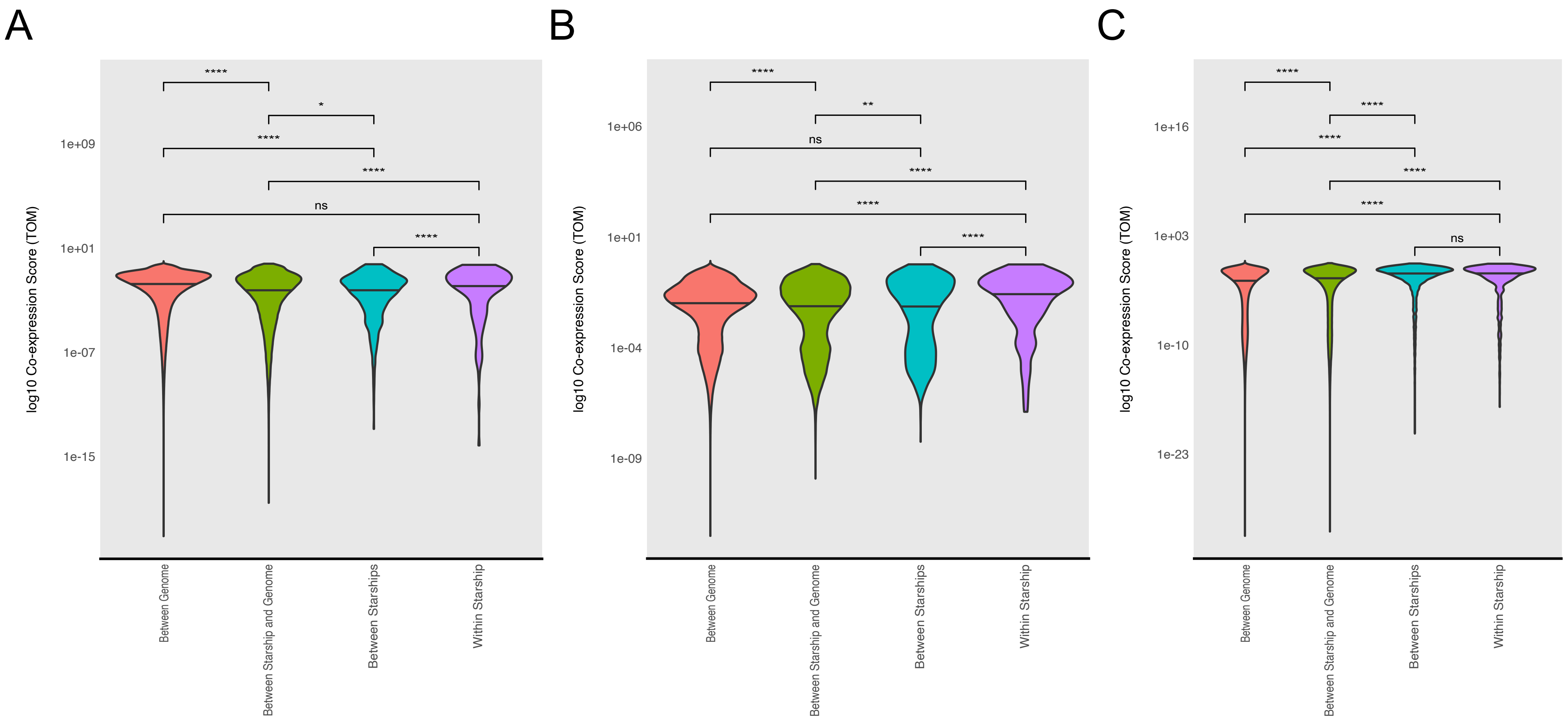
A
B
C
log10 Co-expression Score (TOM)
1e+09
1e+01
1e-07
1e-15
1e+06
1e-04
1e-09
1e+16
1e+03
1e-10
1e-23
****
*
**
ns
Between Genome
Between Starship and Genome
Between Starships
Within Starship

# Figure S16

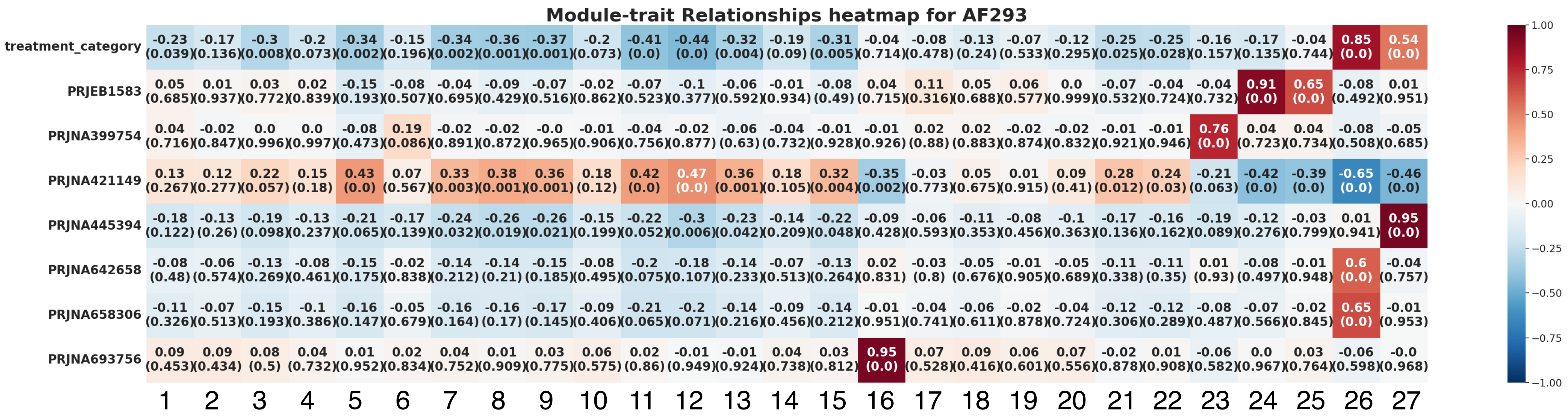

Module-trait Relationships heatmap for AF293
treatment_category: -0.23 (0.039) | -0.17 (0.136) | -0.3 (0.008) | -0.2 (0.073) | -0.34 (0.002) | -0.15 (0.196) | -0.34 (0.002) | -0.36 (0.001) | -0.37 (0.001) | -0.2 (0.073) | -0.41 (0.0) | -0.44 (0.0) | -0.32 (0.004) | -0.19 (0.09) | -0.31 (0.005) | -0.04 (0.714) | -0.08 (0.478) | -0.13 (0.24) | -0.07 (0.533) | -0.12 (0.295) | -0.25 (0.025) | -0.25 (0.028) | -0.16 (0.157) | -0.17 (0.135) | -0.04 (0.744) | 0.85 (0.0) | 0.54 (0.0)
PRJEB1583: 0.05 (0.685) | 0.01 (0.937) | 0.03 (0.772) | 0.02 (0.839) | -0.15 (0.193) | -0.08 (0.507) | -0.04 (0.695) | -0.09 (0.429) | -0.07 (0.516) | -0.02 (0.862) | -0.07 (0.523) | -0.1 (0.377) | -0.06 (0.592) | -0.01 (0.934) | -0.08 (0.49) | 0.04 (0.715) | 0.11 (0.316) | 0.05 (0.688) | 0.06 (0.577) | 0.0 (0.999) | -0.07 (0.532) | -0.04 (0.724) | -0.04 (0.732) | 0.91 (0.0) | 0.65 (0.0) | -0.08 (0.492) | 0.01 (0.951)
PRJNA399754: 0.04 (0.716) | -0.02 (0.847) | 0.0 (0.996) | 0.0 (0.997) | -0.08 (0.473) | 0.19 (0.086) | -0.02 (0.891) | -0.02 (0.872) | -0.0 (0.965) | -0.01 (0.906) | -0.04 (0.756) | -0.02 (0.877) | -0.06 (0.63) | -0.04 (0.732) | -0.01 (0.928) | -0.01 (0.926) | 0.02 (0.88) | 0.02 (0.883) | -0.02 (0.874) | -0.02 (0.832) | -0.01 (0.921) | -0.01 (0.946) | 0.76 (0.0) | 0.04 (0.723) | 0.04 (0.734) | -0.08 (0.508) | -0.05 (0.685)
PRJNA421149: 0.13 (0.267) | 0.12 (0.277) | 0.22 (0.057) | 0.15 (0.18) | 0.43 (0.0) | 0.07 (0.567) | 0.33 (0.003) | 0.38 (0.001) | 0.36 (0.001) | 0.18 (0.12) | 0.42 (0.0) | 0.47 (0.0) | 0.36 (0.001) | 0.18 (0.105) | 0.32 (0.004) | -0.35 (0.002) | -0.03 (0.773) | 0.05 (0.675) | 0.01 (0.915) | 0.09 (0.41) | 0.28 (0.012) | 0.24 (0.03) | -0.21 (0.063) | -0.42 (0.0) | -0.39 (0.0) | -0.65 (0.0) | -0.46 (0.0)
PRJNA445394: -0.18 (0.122) | -0.13 (0.26) | -0.19 (0.098) | -0.13 (0.237) | -0.21 (0.065) | -0.17 (0.139) | -0.24 (0.032) | -0.26 (0.019) | -0.26 (0.021) | -0.15 (0.199) | -0.22 (0.052) | -0.3 (0.006) | -0.23 (0.042) | -0.14 (0.209) | -0.22 (0.048) | -0.09 (0.428) | -0.06 (0.593) | -0.11 (0.353) | -0.08 (0.456) | -0.1 (0.363) | -0.17 (0.136) | -0.16 (0.162) | -0.19 (0.089) | -0.12 (0.276) | -0.03 (0.799) | 0.01 (0.941) | 0.95 (0.0)
PRJNA642658: -0.08 (0.48) | -0.06 (0.574) | -0.13 (0.269) | -0.08 (0.461) | -0.15 (0.175) | -0.02 (0.838) | -0.14 (0.212) | -0.14 (0.21) | -0.15 (0.185) | -0.08 (0.495) | -0.2 (0.075) | -0.18 (0.107) | -0.14 (0.233) | -0.07 (0.513) | -0.13 (0.264) | 0.02 (0.831) | -0.03 (0.8) | -0.05 (0.676) | -0.01 (0.905) | -0.05 (0.689) | -0.11 (0.338) | -0.11 (0.35) | 0.01 (0.93) | -0.08 (0.497) | -0.01 (0.948) | 0.6 (0.0) | -0.04 (0.757)
PRJNA658306: -0.11 (0.326) | -0.07 (0.513) | -0.15 (0.193) | -0.1 (0.386) | -0.16 (0.147) | -0.05 (0.679) | -0.16 (0.164) | -0.16 (0.17) | -0.17 (0.145) | -0.09 (0.406) | -0.21 (0.065) | -0.2 (0.071) | -0.14 (0.216) | -0.09 (0.456) | -0.14 (0.212) | -0.01 (0.951) | -0.04 (0.741) | -0.06 (0.611) | -0.02 (0.878) | -0.04 (0.724) | -0.12 (0.306) | -0.12 (0.289) | -0.08 (0.487) | -0.07 (0.566) | -0.02 (0.845) | 0.65 (0.0) | -0.01 (0.953)
PRJNA693756: 0.09 (0.453) | 0.09 (0.434) | 0.08 (0.5) | 0.04 (0.732) | 0.01 (0.952) | 0.02 (0.834) | 0.04 (0.752) | 0.01 (0.909) | 0.03 (0.775) | 0.06 (0.575) | 0.02 (0.86) | -0.01 (0.949) | -0.01 (0.924) | 0.04 (0.738) | 0.03 (0.812) | 0.95 (0.0) | 0.07 (0.528) | 0.09 (0.416) | 0.06 (0.601) | 0.07 (0.556) | -0.02 (0.878) | 0.01 (0.908) | -0.06 (0.582) | 0.0 (0.967) | 0.03 (0.764) | -0.06 (0.598) | -0.0 (0.968)
1 2 3 4 5 6 7 8 9 10 11 12 13 14 15 16 17 18 19 20 21 22 23 24 25 26 27
1.00
0.75
0.50
0.25
0.00
−0.25
−0.50
−0.75
−1.00
